# Effects of Digital Mindfulness Training for Couples on Psychological Distress and Infant Neuropsychological Development: Randomized Controlled Trial

**DOI:** 10.2196/77260

**Published:** 2025-11-21

**Authors:** Yunxia Tian, Rui Ma, Naixue Cui, Juan Wang, Yongqi Huang, Kaiyan Guo, Xiaodong Liu, Hui Fang, Mengyuan Dong, Caiping Wan, Xuan Zhang, Fenglin Cao

**Affiliations:** 1 School of Nursing and Rehabilitation, Cheeloo College of Medicine, Shandong University Jinan China; 2 School of Nursing, Ningxia Medical University Yinchuan China; 3 Department of Obstetrics and Gynecology, Peking University First Hospital, Ningxia Women and Children’s Hospital (Ningxia Hui Autonomous Region Maternal and Child Health Hospital) Yinchuan China; 4 Affiliated Mental Health Center and Hangzhou Seventh People’s Hospital and School of Brain Science and Brain Medicine, Zhejiang University School of Medicine Hangzhou China; 5 Neonatal Intensive Care Unit, Peking University First Hospital, Ningxia Women and Children’s Hospital (Ningxia Hui Autonomous Region Maternal and Child Health Hospital) Yinchuan China; 6 The second hospital of Shandong University Jinan China

**Keywords:** couple intervention, digital interventions, infant, mindfulness-based interventions, neuropsychological development, pregnancy, psychological distress, randomized controlled trial

## Abstract

**Background:**

Maternal psychological distress during pregnancy is associated with unfavorable infant outcomes. Although mindfulness-based interventions can effectively alleviate such distress, barriers often limit access to face-to-face interventions. Psychological distress among partners is also common during pregnancy and affects maternal mental health and the parent–child relationship. However, corresponding research remains scarce.

**Objective:**

We evaluated the effectiveness of a digital mindfulness-based intervention for expectant parents (dMBI-EP) in reducing parental psychological distress and improving infant neuropsychological performance.

**Methods:**

We recruited 160 couples in this randomized controlled trial who were expecting their first child, with the pregnant women at 12-20 weeks of gestation. The couples were randomized into a dMBI-EP group (n=80) receiving regular perinatal care plus a 6-week intervention delivered through a WeChat mini-program and a control group (n=80) receiving regular perinatal care only. The primary outcomes were parental psychological distress symptoms (depression, anxiety, and perceived stress) assessed at baseline (T1), 2 weeks after completion of the intervention (T2), and 6 weeks post partum (T3). Secondary outcomes included other parental psychological outcomes (fatigue, sleep problems, marital intimacy, and parental–fetal bonding) assessed at T1 and T2, and infant neuropsychological outcomes assessed at T3.

**Results:**

The study included 160 pregnant women (mean age 26.89, SD 2.78 years) and their 160 partners (mean age 29.01 years, SD 3.21). For pregnant women in the intervention group, the average levels of depression, anxiety, and perceived stress shifted from 9.28 (SD 5.50), 5.04 (SD 3.39), and 14.85 (SD 5.46) to 6.40 (SD 4.64), 3.90 (SD 3.00), and 11.50 (SD 5.76), respectively. In the control group, the corresponding levels shifted from 10.04 (SD 4.71), 5.84 (SD 3.70), and 14.91 (SD 5.08) to 9.84 (SD 5.86), 5.66 (SD 4.14), and 15.53 (SD 6.23). The mean between-group differences for depression, anxiety, and perceived stress were –3.56 (Cohen *d*=0.65; *P*<.001), –1.80 (Cohen *d*=0.49; *P*=.002), and –4.01 (Cohen *d*=0.67; *P*<.001) at T2, respectively. For intervention group partners, the average levels of depression, anxiety, and perceived stress shifted from 6.94 (SD 5.45), 3.00 (SD 3.20), and 13.46 (SD 5.98) to 5.39 (SD 4.69), 3.11 (SD 3.41), and 11.53 (SD 7.96), respectively, whereas the corresponding levels shifted from 6.13 (SD 4.38), 3.65 (SD 3.09), and 12.95 (SD 5.34) to 7.28 (SD 5.39), 4.17 (SD 3.81), and 12.67 (SD 6.04) in the control group. The mean between-group difference in depression at T2 was –1.95 (Cohen *d*=0.38; *P*=.006). Moreover, the average scores of infants’ activity level, approach, intensity of reaction, quality of mood, distractibility, and adaptability at 6 weeks post partum were significantly higher in the control than in the intervention group (2-tailed *t*_122_=2.330-5.124; all *P*<.05), with Cohen *d* ranging from 0.42 to 0.92.

**Conclusions:**

The dMBI-EP reduced the scores of psychological distress in couples, with potentially sustained benefits extending into the early postpartum period, and may contribute to more favorable infant neuropsychological development outcomes.

**Trial Registration:**

Chinese Clinical Trial Register ChiCTR2200059598; https://www.chictr.org.cn/showproj.html?proj=162809

## Introduction

Pregnancy and childbirth, particularly the gestation and birth of the first child, represent pivotal life transitions and developmental milestones within the family life cycle [1]. In particular, the antenatal period imposes unique psychological demands on couples, as pregnancy-specific physiological changes (eg, hormonal fluctuations and somatic alterations) intersect with the developmental task of preparing for parental identity formation. Depression, anxiety, and stress are common psychological challenges experienced during pregnancy that affect approximately 10% of pregnant women [2]. Compared with pregnant women, the psychological distress experienced by their partners during the same period has often been overlooked. Indeed, the literature reports that the prevalence of psychological distress in partners during pregnancy is comparable to that in pregnant women [2]. This oversight has critical implications, as research has shown that paternal distress not only disrupts marital functioning but also affects maternal mental health and subsequent parent–child relationships [3].

Although numerous interventions have been shown to alleviate psychological distress in pregnant women, those specifically targeting partners and interventions that simultaneously involve both members of the couple remain rare. The absence of programs designed to engage pregnant women and their partners represents a critical gap, as research has demonstrated that psychological distress is interdependent. Such distress is shared between both members of the couple dyad, potentially through “emotional contagion” or a multilayered process in which stimuli from one individual elicit an emotional or a behavioral response in the other (ie, the “common fate” model) [4]. These findings underscore the need for a paradigm shift toward integrating dyadic assessments into perinatal screening protocols and developing a partner-inclusive treatment framework.

Mindfulness-based interventions (MBIs) are designed to foster awareness of individuals’ present-moment experiences and to develop an orientation of openness and acceptance toward these experiences [5]. MBIs are widely recognized as effective psychological approaches in both clinical and nonclinical populations, including pregnant women [6-8]. Multiple systematic reviews and meta-analyses [9-11] indicate that MBIs help alleviate depression, anxiety, and perceived stress in pregnant women and have long-term preventive effects on postpartum depression and perceived stress [12]. However, MBIs for both partners primarily focus on couples coping with chronic illnesses such as cancer [13]. To the best of our knowledge, only 2 randomized controlled trials (RCTs) have used mindfulness interventions with couples. One study used a mindfulness-based childbirth and parenting program that integrated mindfulness training with childbirth and parenting education [14]. However, it specifically targeted couples in which the pregnant woman feared childbirth, with childbirth-related outcomes as the primary focus. Another study focused on couples’ relationship satisfaction and recruited couples with excessively high levels of relationship satisfaction. In addition, the sample size was small [15]. All these factors limited the representativeness of the 2 studies. Thus, it remains unclear whether mindfulness-based dyadic interventions are suitable for couples.

One of the challenges of traditional MBIs is the requirement to attend in-person classes that last 6-8 weeks. This becomes even more difficult when both partners in a couple are expected to participate, because their conflicting schedules and responsibilities often make coordination challenging [16]. Two recent systematic reviews and meta-analyses showed that emerging digital mindfulness interventions can significantly reduce maternal prenatal depressive and anxiety symptoms [17] and are also effective in preventing postpartum depression in healthy pregnant women [18]. The digital approach offers an innovative solution that can effectively overcome these obstacles. It provides individuals with the flexibility to engage in therapy at their convenience, breaking free from the limitations of time and location while also helping to reduce the stigma often associated with seeking psychological support [19]. These benefits are particularly significant in relatively resource-limited and less developed areas [20], such as western China.

WeChat (Tencent) is the most widely used smartphone application for instant communication, social interaction, and information dissemination in China and is regarded as one of the leading social networks globally [21]. Its mini-program function enables simpler development of health intervention programs, reduces costs, and delivers a more rapid, convenient, and enriched user experience [22,23]. Thus, the primary aim of this study was to explore the effect of a digital mindfulness-based intervention for expectant parents (dMBI-EP) through a WeChat mini-program on their psychological stress responses.

The early neuropsychological development of offspring is equally significant to the parents’ mental health during pregnancy. Neuropsychological development refers to the developmental process of the brain structure and function, as well as behavioral performance under the dynamic interaction of biological, environmental, and cultural factors, involving adaptive changes in multiple dimensions such as cognition, language, motor skills, and social-emotional functions [24]. Temperament refers to biologically-based, relatively stable individual differences in emotional reactivity and regulation and other behavioral tendencies [25] that are observable at birth [26]. Infant neuropsychological studies often include infant temperament as a characteristic of behavioral performance and incorporate it into the measurement framework [27,28]. Moreover, temperament serves as a crucial early predictor of an individual’s later-stage psychopathology and developmental behavior disorders, thus holding significant research value [29,30]. Two previous reviews have highlighted a relationship between maternal prenatal mental health and the difficult-to-care-for temperament of infants [31,32]. Two systematic reviews and meta-analyses published in 2024 showed significant associations between maternal prenatal depression and poorer cognitive, language, and motor development in offspring [33]. Moreover, parental prenatal mood and anxiety disorders were significantly associated with an increased risk of neurodevelopmental disorders in children [34]. Given the negative consequences of couples’ prenatal psychological distress, effective primary preventive interventions are crucial. Pregnancy, that is, the early developmental period of offspring, is a window of opportunity in which early preventive interventions may not only reduce the burden and alleviate the suffering resulting from couples’ prenatal psychological distress but also leverage neuroplasticity to optimize neurodevelopmental trajectories across the lifespan [35]. Therefore, the second aim of this study was to explore the potential impact of dMBI-EP on offspring’s neuropsychological development.

## Methods

### Ethical Considerations

The study protocol underwent review and received approval from the Ethics Committee of Shandong University School of Nursing and Rehabilitation (2022-R-61). Informed consent was obtained from all participants in the study. The trial was registered at the Chinese Clinical Trial Registry (ChiCTR2200059598). All data and information from participants were strictly confidential and safeguarded. Participants were not paid for their participation in the study, except for small gifts such as baby and maternity tissues as a form of periodic incentive. All participants had access to free and convenient WeChat-based antenatal health consultations provided by trained assistants who were unaware of the study allocation. This study adhered to the standard guidelines for RCTs and is reported according to the CONSORT-EHEALTH (Consolidated Standards of Reporting Trials of Electronic and Mobile Health Interventions and Online Telehealth) guidelines for reporting eHealth and mHealth interventions (Multimedia Appendix 1).

### Study Design and Participant Eligibility

This study was a 2-arm RCT conducted with couples during pregnancy who attended routine obstetric examinations at the outpatient centers of a specialized hospital in Yinchuan City, Ningxia Hui Autonomous Region, from June 2022 to December 2023. Follow-up was completed in September 2024, with a total of 408 couples recruited for the study.

The inclusion criteria for pregnant women were as follows: (1) age ≥18 years, (2) singleton pregnancy, (3) expecting their first childbirth, (4) gestational age 12-20 weeks, (5) proficiency in reading and writing Chinese, and (6) no participation in any other psychological intervention or assignment to a waiting group. The exclusion criteria were as follows: (1) severe somatic or mental disorders before or during pregnancy, (2) serious comorbidities or complications during pregnancy, (3) long-term separation from their husband, and (4) previous experience with mindfulness and meditation practices (eg, yoga).

For partners, the inclusion criteria were: (1) age ≥18 years, (2) expecting their first child, (3) proficiency in reading and writing Chinese, (4) no participation in any other psychological interventions or assignment to a waiting group, and (5) identified by the pregnant woman as her current partner. The exclusion criteria for partners were: (1) severe somatic or mental disorders, (2) long-term separation from their wife, and (3) previous experience with mindfulness and meditation practices (eg, yoga).

In our initial research design, we planned to collect neonates’ feces within the first 48 hours after birth to analyze the potential impact of dMBI-EP on fetal gut microbiota. Consequently, pregnant women with HIV infection, hepatitis C infection, immunosuppressive diseases, or those who had undergone gastrointestinal surgery within the past 5 years (excluding cholecystectomy and appendectomy) were initially intended to be excluded. Nevertheless, due to insufficient project funding, we abandoned this part of the study and did not apply the corresponding exclusion criteria. Revocation of these exclusion criteria did not influence this study.

### Sample Size

The sample size was calculated using G*Power (Heinrich Heine University Düsseldorf) software. Previous studies [22,36,37] have shown a medium effect size for anxiety and depression after mindfulness interventions during pregnancy, while self-help interventions for these conditions yielded small to medium effects. Based on a medium effect size (Cohen *d*=0.50), a sample size of 128 was needed to achieve 80% power with a 2-sided *P*<.05. To account for a 20% attrition rate, 160 participants were recruited.

### Procedures

Pregnant women between 12 and 20 weeks of gestation who attended routine prenatal check-ups with their partners were enrolled in the study (baseline, T1). Randomization was carried out using a computerized random number generator, with the numbers concealed and assigned sequentially to participants. Eligible couples who consented to participate were randomly assigned in a 1:1 ratio to either the intervention group (regular prenatal care plus mindfulness intervention) or the control group (regular prenatal care only).

### The Intervention: dMBI-EP

Our intervention was implemented through a WeChat mini-program, titled “Mindfulness Brightens Pregnant Couples’ Moods.” The intervention program drew on the contents of mindfulness-based stress reduction [38] and mindfulness-based childbirth and parenting [39]. A series of specific and targeted adjustments was made to ensure that the intervention components and procedures remained essentially unchanged. These modifications encompassed the following aspects: (1) based on previous research [40], the intervention duration and number of modules were shortened to 6 weeks; (2) the characters were set as ordinary young Chinese couples, either in cartoon form or in real-life appearance; (3) the language was set as the widely used Mandarin Chinese; (4) animations were created to achieve visualization using appropriate pictures, symbols, diagrams, and metaphors consistent with the text content, and Chinese subtitles were included; and (5) considering the participation of couples from different ethnic groups, religious elements were carefully avoided in the character images, language, music, and animation presentations.

The dMBI-EP consists of six 1-week modules, each including a thematic session and 6 audio-guided home practice sessions. On the first day of each module, couples were required to watch an animated video, which was 12-20 minutes long. The video presented theoretical content related to mindfulness designed for couples, and some of the videos (covering all 6 weeks) included mindfulness practices demonstrated by our team members. Over the remaining 6 days of the week, couples were encouraged to engage in both formal (audio-guided) and informal mindfulness practices (with mindfulness practices integrated into daily activities) at home. Each module incorporated content related to the partner, which was presented not in isolation but based on the “commonalities, emotional bond, and interaction” with the pregnant woman, emphasizing the consensus, empathy, and collaboration between couples.

For example, in the thematic lessons, in the first week, we introduced “Prenatal Psychological Stress of Couples,” explaining its causes, symptoms, and negative impacts, and emphasized equal attention to the partners’ prenatal psychological stress. In the fourth week, parents were taught “How to Interact With the Fetus During Pregnancy,” including “Mindful Touching of Fetal Movement” and “Mindful Listening to the Fetal Heart With the Help of a Fetal Heart Monitor.” In the fifth week, “Mindful Stretching” and “Ice-Holding Exercise” were explained and demonstrated to the couple, requiring their cooperation. In the sixth week, the couple learned “Learning Mindful Eye Contact, Hugging, Listening/Speaking to Support Each Other.” This module aimed to integrate mindfulness into daily life, prompting couples to purposefully pay attention to, respond to, and be attuned to each other in their daily living.

**Figure 1:**
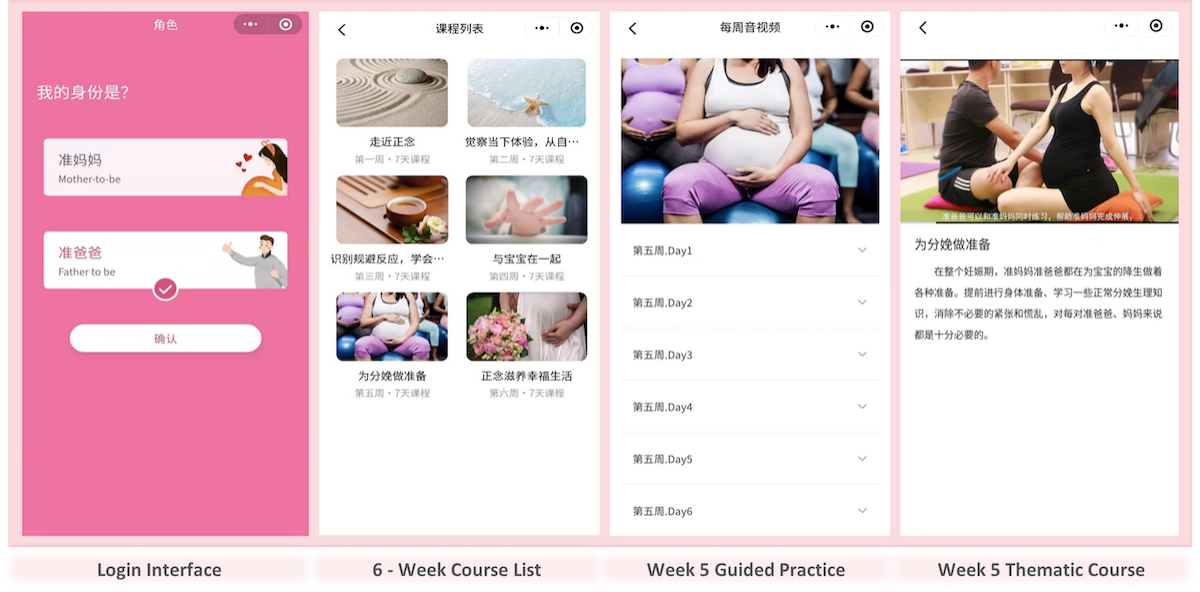
The interface display of the WeChat mini-program “Mindfulness Brightens Pregnant Couples’ Moods.”.

In the audio-guided home practice section, there was detailed guidance on mindfulness exercises that specifically involved couple collaboration and interaction (Multimedia Appendix 2). Couples were expected to study and practice together, and the mini-program tracked users’ logins and learning activities. While each partner’s mini-program was linked, couples shared one account for convenience, with the program asking them to confirm whether they were studying and practicing together. Researchers sent standardized WeChat reminders when the first day’s content of each module was unlocked: “Hello! Week X’s courses have been unlocked. Remember to attend classes and practice mindfulness with your partner daily during your free time.” The Coach Guiding sessions were held at the end of the second and sixth weeks, each lasting 15-20 minutes. Conducted as group WeChat video conferences led by project team personnel, these sessions included a Q&A to address participants’ questions, along with encouragement and sharing. The session at the end of the sixth week also included a summary. Multimedia Appendix 2 depicts the program outline, and Figure 1 presents a screenshot of the mini-program.

### Outcomes

The primary outcomes were couples’ psychological stress response symptoms during pregnancy, including depression, anxiety, and perceived stress, assessed at baseline (12-20 weeks of gestation, T1), within 2 weeks of completion of the intervention (approximately 20-28 weeks of gestation, T2), and 6 weeks post partum (T3). Secondary outcomes included other psychological outcomes of couples (including fatigue, sleep issues, prenatal attachment, and perceived partner responsiveness) at T1 and T2, and infant neuropsychological development assessed by wives at T3.

### Measures

The Edinburgh Postpartum Depression Scale (EPDS) [41] consists of 10 items, each scored on a 4-point scale (0-3), with total scores ranging from 0 to 30. Higher scores indicate more severe symptoms. A sum score of 9 or higher is used to screen for mild or possible depression symptoms [42]. It is a validated tool for assessing prenatal depression [43], including the Chinese version [44].

The 7-item Generalized Anxiety Disorder Questionnaire (GAD-7) [45] is a widely used tool, applicable to clinical practice and research. Each item is scored on a scale from 0 to 3, and the total scores range from 0 to 21, with higher scores signifying greater anxiety levels. A score of 5 or higher on the GAD-7 may indicate possible anxiety symptoms [46]. The Chinese version of the GAD-7 has shown good psychometric properties [47].

The 10-item Perceived Stress Scale (PSS-10) [48] is scored on a scale from 0 to 4 for each item, with total scores ranging from 0 to 40. Higher scores reflect higher perceived stress. A score of 14 or above suggests moderate to high levels of perceived stress [49]. The Chinese version has shown reliability and validity [50].

The Chalder Fatigue Scale, which was used to assess the severity of physical and mental fatigue [51], consists of 14 items. Each item has 2 response options (yes or no), and the scale yields total scores ranging from 0 to 14. Higher scores indicate higher chronic fatigue levels. The Chinese version of the Chalder Fatigue Scale-14 has demonstrated satisfactory reliability and validity [52].

We used the 8-item Athens Insomnia Scale (AIS) to assess sleep problems. This validated instrument quantifies sleep difficulties according to the International Classification of Diseases, 10th Revision diagnostic criteria [53]. The AIS is rated on a 0-3 scale, and total scores range from 0 to 24, with higher scores indicating poorer sleep quality. The Chinese AIS version has also been shown to be reliable and valid [54].

The 12-item Perceived Partner Responsiveness Scale [55] was used to assess marital intimacy. Each item is rated on a 7-point Likert scale (1-7). The average of all 12 items should be calculated, with higher scores indicating greater perceived partner responsiveness by the respondent. In terms of shared experiences and coping with stress, this scale may be more applicable than one-way relationship measurement instruments [56]. The Chinese version of the Perceived Partner Responsiveness Scale is a valid and reliable tool for measuring adult partners’ intimate relationships [57].

The 20-item Five Facet Mindfulness Questionnaire [58] was used to assess mindfulness level. Each item can be rated on a 1-5 scale, from “strongly disagree” to “strongly agree.” The total score ranges from 20 to 100, and higher scores indicate higher mindfulness level.

Parental bonding to the fetus was measured using the Maternal Antenatal Attachment Scale/Paternal Antenatal Attachment Scale (MAAS/PAAS) [59,60]. The MAAS comprises 19 items, while the PAAS contains 16 items. All items in the MAAS/PAAS are rated on a 5-point Likert scale (1-5). Consequently, the total scores of the MAAS range from 19 to 95, while those of the PAAS range from 16 to 80. Higher scores indicate a higher level of bonding with the fetus. The Chinese versions of MAAS and PAAS are valid and reliable tools for measuring parental bonding to the fetus [61,62].

Infants’ neuropsychological development was measured using the Age and Stages Questionnaire–Third Edition [63]. The assessment consists of 30 items rated on a 3-point Likert scale, covering 5 domains that evaluate the development of key functional areas in infants: communication, gross motor, fine motor, problem-solving, and personal-social. For each domain, the total score ranges from 0 to 60, with lower scores indicating poorer developmental behaviors.

Infant temperament was measured using the Early Infancy Temperament Questionnaire [64,65]. This 76-item, 9-dimension parent questionnaire is designed to evaluate temperament characteristics in infants aged 1-4 months. The 9 dimensions include activity level, rhythmicity, approach, intensity of reaction, quality of mood, attention span, distractibility, threshold of reaction, and adaptability. Each item is rated on a 6-point frequency scale, ranging from “almost never” to “almost always.” Higher scores on each dimension indicate greater activity, irregular rhythm, easier withdrawal, stronger reactions, a more negative mood, shorter persistence, greater distractibility, a lower reaction threshold, and slower adaptation, indicating a more negative infant temperament and greater caretaking difficulty [66].

### Demographic Characteristics, Pregnancy Conditions, and Neonatal Outcomes

The sociodemographic questionnaire covered age, ethnic group, education level, residence, monthly family income, whether the couple resided together, whether the pregnancy was planned, and specific pregnancy-related details of the pregnant woman, such as gestational age, adverse pregnancy history (including abortion or induced labor), and prenatal complications (eg, anemia, subhypothyroidism, gestational diabetes, pregnancy-induced hypertension, gestational heart disease, threatened abortion, and placenta previa). Neonatal outcome information, including birth weight and gestational age at delivery, was obtained by interviewing the parents post partum.

### Collection of Outcome Data

The outcome data were collected using paper-based and electronic questionnaires. The baseline assessment was mainly conducted using paper-based questionnaires. During subsequent assessments, we sent electronic questionnaire links using the WenjuanXing platform through WeChat. For participants who preferred not to complete the electronic version, supplementary paper questionnaires with identical content were made available.

### Adherence

Module completion was defined as completing at least 1 thematic session and 3 of the 6 formal practice sessions per week. Intervention compliance was defined as completing at least 3 modules, including video courses and home practices. The WeChat mini-program supported participants’ daily learning and formal practice. The intervention completion rate was calculated as the proportion of participants who completed the intervention among those in the intervention group.

### Statistical Analysis

Data were expressed as mean (SD), n (%), and 95% CI. A series of chi-square tests, Fisher exact tests, and 2-tailed *t* tests were performed to assess demographic differences between the intervention and control groups. Linear regression analysis was used to assess the relationship between the frequency of mindfulness learning, practice time, and psychological distress indices. A generalized estimated equation (GEE) was used to assess the impact of the intervention on psychological stress responses. An independent-samples *t* test was used to examine the effect of the intervention on the infants’ neuropsychological development. The false discovery rate with Benjamini-Hochberg correction was used to adjust the *P* values.

Our RCT analysis adhered to the intention-to-treat principle. All randomly assigned participants were included in the final statistical analysis to minimize bias caused by dropouts or insufficient compliance. The GEE can handle correlation issues in longitudinal follow-up data and, to some extent, enables robust estimation with missing outcome data. In this study, there were no missing baseline outcome variables; therefore, all participants were included in the analysis. For individual missing cases during the follow-up period, the GEE method could still perform effective estimation, thus ensuring the robustness of the results.

We conducted a partial correlation analysis to explore the relationship between infant temperament dimensions and parental psychological distress at T2 while controlling for T1 distress. Using the SPSS PROCESS plugin (Model 4; IBM Corp), we examined whether the intervention affected infants’ neuropsychological development by mitigating couples’ psychological distress during pregnancy. The bias-corrected nonparametric percentile bootstrapping method (n=5000) was used to test the significance of the mediation effect, with a 95% CI not including 0 indicating a significant mediation effect. As the mediation analysis was exploratory, no correction for multiplicity was performed.

## Results

### Sample Characteristics

**Figure 2:**
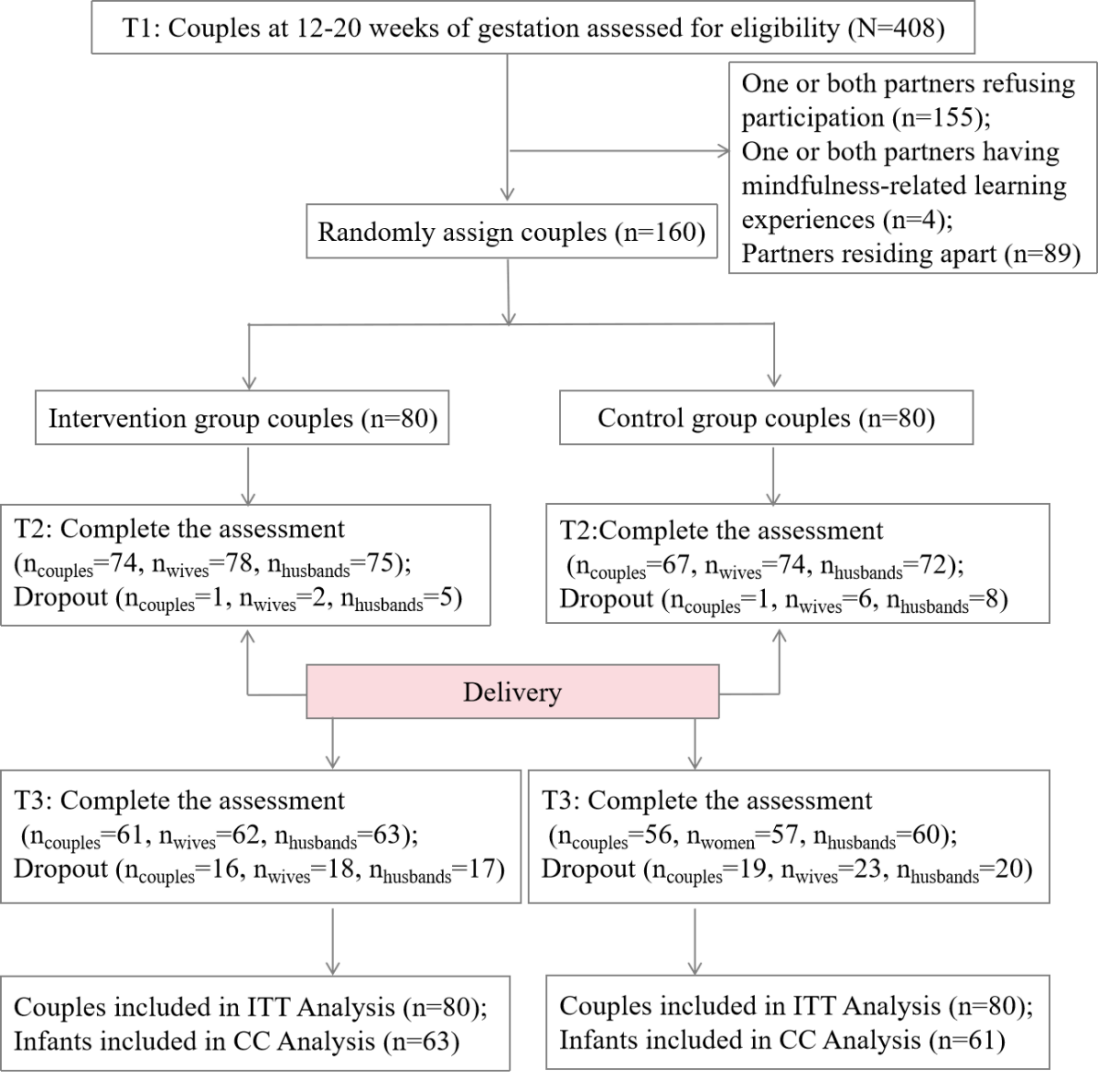
Participant flow diagram. CC: complete case; ITT: intention-to-treat; T1: baseline (12-20 weeks of gestation); T2: 2 weeks after completion of the intervention (approximately 20-28 weeks of gestation); T3: 6 weeks postpartum.

We initially approached 408 pregnant couples between 12 and 20 weeks of gestation and obtained a final sample of 160 couples (Figure 2). Among the 160 couples, 141 (88.1%) completed the assessment after the intervention (T2), and 2 couples (one each from the intervention and control groups) were lost to follow-up. In addition, 17 individuals (wives or husbands) from different couples were lost to follow-up. Specifically, 2 wives and 5 husbands dropped out of the intervention group, whereas 6 wives and 8 husbands dropped out of the control group. The overall dropout rate was 5% (8/160) for wives and 8.13% (13/160) for husbands.

At T3, 117 (73.1%) couples completed the assessment. In total, 16 couples dropped out of the intervention group and 19 dropped out of the control group. In addition, 8 individuals (wives or husbands) from different couples were lost to follow-up. Specifically, 18 wives and 17 husbands dropped out of the intervention group, whereas 23 wives and 20 husbands dropped out of the control group. This resulted in an overall dropout rate of 25.6% (41/160) for wives and 23.1% (37/160) for husbands.

Table 1 presents the sample characteristics at baseline (T1). Pregnant women’s mean age was 26.89 (SD 2.78) years, and the mean gestational age was approximately 14.11 (SD 2.55) weeks. Partners’ mean age was 29.01 (SD 3.21) years. There were no single pregnant women among the participants. The mean gestational age at delivery was 38.92 (SD 1.48) weeks, and the mean infant birth weight was 3261.68 (SD 465.71) grams.

**Table 1:** Demographic characteristics and pregnancy-related conditions of couples. A currency exchange rate of Yuan ¥1=US $0.1401 is applicable.

Characteristic				Overall (N=160)		Control group (n=80)		Intervention group (n=80)
**Pregnant women**								
	Age (in years), mean (SD)			26.89 (2.78)		27.05 (2.67)		26.74 (2.90)
	**Nationality, n (%)**							
		Han	135 (84.4)		68 (85.0)		67 (83.8)	
		National minority	25 (15.6)		12 (15.0)		13 (16.3)	
	**Education, n (%)**							
		High school or less	26 (16.3)		14 (17.5)		12 (15.0)	
		Junior college	57 (35.6)		30 (37.5)		27 (33.8)	
		Undergraduate or above	77 (48.1)		36 (45.0)		41 (51.3)	
	Gestational age (in weeks), mean (SD)			14.11 (2.55)		13.79 (2.34)		14.43 (2.73)
	**Adverse pregnancy history (yes), n (%)**							
		Yes	30 (18.8)		18 (22.5)		12 (15.0)	
		No	130 (81.3)		62 (77.5)		68 (85.0)	
	**Complications during pregnancy, n (%)**							
		Yes	123 (76.9)		21 (26.3)		16 (20.0)	
		No	37 (23.1)		59 (73.8)		64 (80.0)	
	**Current employment status, n (%)**							
		Yes	126 (78.8)		63 (78.8)		63 (78.8)	
		No	34 (21.3)		17 (21.3)		17 (21.3)	
	Prepregnancy BMI (kg/m^2^), mean (SD)			21.77 (3.42)		21.99 (3.30)		21.56 (3.53)
	Current BMI (kg/m^2^), mean (SD)			22.40 (3.49)		22.63 (3.38)		22.18 (3.61)
**Husbands**								
	Age (in years), mean (SD)			29.01 (3.21)		28.90 (2.60)		29.13 (3.73)
	**Nationality, n (%)**							
		Han	135 (84.4)		70 (87.5)		65 (81.3)	
		National minority	25 (15.6)		10 (12.5)		15 (18.8)	
	**Education, n (%)**							
		High school or less	33 (20.6)		17 (21.3)		16 (20.0)	
		Junior college	57 (35.6)		28 (35.0)		29 (36.3)	
		Undergraduate or above	70 (43.8)		35 (43.8)		35 (43.8)	
	**Current employment status, n (%)**							
		Yes	158 (98.8)		79 (98.8)		79 (98.8)	
		No	2 (1.3)		1 (1.3)		1 (1.3)	
	Current BMI (kg/m^2^), mean (SD)			24.19 (3.50)		24.27 (3.48)		24.12 (3.55)
**Conditions of couples**								
	**Residence, n (%)**							
		Urban	131 (81.9)		63 (78.8)		68 (85.0)	
		Rural	29 (18.1)		17 (21.3)		12 (15.0)	
	**Monthly household income (¥), n (%)**							
		<5000	31 (19.4)		14 (17.5)		17 (21.3)	
		5000-8999	83 (51.9)		46 (57.5)		37 (46.3)	
		≥9000	46 (28.8)		20 (25.0)		26 (32.5)	
	**Both partners intended this pregnancy, n (%)**							
		Yes	110 (68.8)		58 (72.5)		52 (65.0)	
		No	50 (31.3)		22 (27.5)		28 (35.0)	
	**Mode of pregnancy, n (%)**							
		Natural pregnancy	159 (99.4)		80 (100.0)		79 (98.8)	
		Assisted pregnancy	1 (0.6)		0 (0)		1 (1.3)	
**Characteristics of infants, mean (SD)**								
	Gestational age at birth (in weeks), mean (SD)			38.92 (1.48)		39.15 (1.47)		38.69 (1.46)
	Birth weight (g), mean (SD)			3261.68 (465.71)		3329.00 (458.87)		3192.22 (466.10)

In addition, the findings revealed that among 160 couples, at least 1 partner in 148 (92.5%) couples tested positive for at least 1 of the 3 primary outcomes (EPDS≥9 [42], GAD-7≥5 [46], and PSS-10≥14 [49]). Specifically, 134 (83.8%) wives and 106 (66.3%) husbands tested positive for at least 1 of these 3 outcomes (Multimedia Appendix 3).

As shown in Multimedia Appendices 4 and 5, there were no statistically significant differences in the distribution of treatment allocation, general demographic data, and baseline psychological distress indicators between dropout and nondropout samples of pregnant women and partners at T2 and T3 (*P*>.05). For partners, there were no statistically significant differences in the distribution of all baseline psychological outcomes and mindfulness levels between the dropout and nondropout samples at T2 and T3 (*P*>.05). However, pregnant women who dropped out at T2 had higher mindfulness and maternal-fetal bonding levels than those who remained.

### Adherence

In the intervention group, 45 (56.25%) couples completed theme courses for at least 3 modules, but only 37 (46.25%) completed theme courses and formal home exercises for 3 weeks or more, meeting the compliance criteria for this study. Specifically, pregnant women completed the theme courses and home exercises for an average of 3.36 (SD 2.28) weeks and 2.41 (SD 2.23) weeks, respectively, and an average of 15.09 (SD 12.23) sessions in total (range 0-42 sessions), while partners completed them for an average of 3.08 (SD 2.31) weeks and 1.85 (SD 1.87) weeks, respectively, and an average of 12.50 (SD 11.32) sessions in total (range 0-41 sessions). A total of 41 (51.25%) pregnant women and 37 (46.25%) partners met the study compliance criteria. Approximately 74 (92.75%) couples received the first coaching guidance session, and 43 (53.75%) received the second session.

Considering that the frequency of learning and practice may affect the psychological stress response and mindfulness levels, we conducted a series of linear regression analyses. The results revealed that the total number of class sessions for learning mindfulness and formal home practice was not significantly associated with psychological outcomes or mindfulness levels at T2 (in all analyzed models, *P*>.05, and the 95% CI for the regression coefficient included 0) (Multimedia Appendix 6).

### Effectiveness on Primary Outcomes

**Figure 3:**
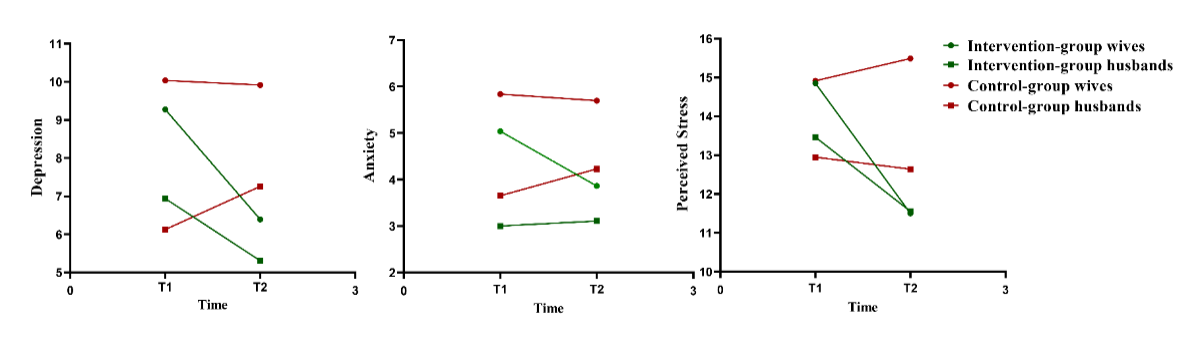
Changes in psychological distress of couples over time in the intervention group and the control group. T1: baseline (12-20 weeks of gestation); T2: 2 weeks after completion of the intervention (approximately 20-28 weeks of gestation).

As shown in Table 2 and Figure 3, the results for pregnant women indicated a significant time×group interaction for symptoms of depression (Wald *χ*²_1_=10.1; *q*=.002) and perceived stress (Wald *χ*²_1_=18.6; *q*<.001). Changes in depression and perceived stress symptoms from preintervention to postintervention differed significantly between the intervention and control groups. In the intervention group, depression and perceived stress symptoms at T2 were significantly lower compared with the control group, with Cohen *d* values of 0.49 and 0.67, respectively. The outcomes imply an nonsignificant time×group interaction for symptoms of anxiety (Wald *χ*²_1_=2.5; *q*=.11). Nevertheless, the anxiety levels of pregnant women in the intervention group were lower than those of pregnant women in the control group.

**Table 2:** Overall test results and between-group differences in couples’ psychological distress from the generalized estimating equations analysis. T1: baseline (12-20 weeks of gestation); T2: 2 weeks after completion of the intervention (approximately 20-28 weeks of gestation).

Outcomes			Control group, mean (SD)		Intervention group, mean (SD)		Estimated mean difference, (95% CI)		Cohen *d* (95% CI)		Group×time				
											*Wald χ*^2^ (*df*)		*P* value		*q* value (FDR^a^ adjusted)
**Maternal depression**															
	T1	10.04 (4.71)		9.28 (5.50)		–0.76 (–2.34 to 0.81)		N/A^b^		10.1 (1)		.001		.002	
	T2	9.84 (5.86)		6.40 (4.64)		–3.56 (–5.22 to –1.90)		0.65 (0.32 to 0.98)		N/A		N/A		N/A	
**Paternal depression**															
	T1	6.13 (4.38)		6.94 (5.45)		0.81 (–0.71 to 2.34)		N/A		9.6 (1)		.002		.006	
	T2	7.28 (5.39)		5.39 (4.69)		–1.95 (–3.55 to –0.34)		0.38 (0.05 to 0.70)		N/A		N/A		N/A	
**Maternal anxiety**															
	T1	5.84 (3.70)		5.04 (3.39)		–0.80 (–1.89 to 0.29)		N/A		2.5 (1)		.11		0.11	
	T2	5.66 (4.14)		3.90 (3.00)		–1.80 (–2.93 to –0.66)		0.49 (0.17 to 0.81)		N/A		N/A		N/A	
**Paternal anxiety**															
	T1	3.65 (3.09)		3.00 (3.20)		–0.65 (–1.62 to 0.32)		N/A		0.7 (1)		.42		0.42	
	T2	4.17 (3.81)		3.11 (3.41)		–1.14 (–2.30 to 0.01)		0.29 (–0.03 to 0.62)		N/A		N/A		N/A	
**Maternal perceived stress**															
	T1	14.91 (5.08)		14.85 (5.46)		–0.06 (–1.69 to 1.56)		N/A		18.7 (1)		<.001		<.001	
	T2	15.53 (6.23)		11.50 (5.76)		–4.01 (–5.89 to –2.14)		0.67 (0.34 to 1.00)		N/A		N/A		N/A	
**Paternal perceived stress**															
	T1	12.95 (5.34)		13.46 (5.98)		0.51 (–1.23 to 2.26)		N/A		1.8 (1)		.18		.26	
	T2	12.67 (6.04)		11.53 (7.96)		–1.08 (–3.33 to 1.17)		0.16 (–0.16 to 0.48)		N/A		N/A		N/A	

^a^FDR: false discovery rate.

^b^N/A: Not available.

For partners, the results indicated a significant time×group interaction for symptoms of depression (Wald *χ*²_1_=9.6; *q*=.006). Changes in depressive symptoms from preintervention to postintervention differed significantly between the intervention and control groups. Depression symptoms at T2 were significantly lower in the intervention group than in the control group with Cohen *d*=0.38. The results also showed a nonsignificant time×group interaction for symptoms of anxiety (Wald *χ*²_1_=0.7; *q*=.42) and perceived stress (Wald *χ*²_1_=1.8; *q*=.26). Changes in anxiety and perceived stress symptoms did not differ significantly between the intervention and control groups from preintervention to postintervention.

### Effectiveness on Secondary Outcomes

#### Postpartum Psychological Distress

The results for wives at 6 weeks post partum (T3) revealed a significant time×group interaction for symptoms of depression (Wald *χ*²_1_=13.5; *q*=.002) and perceived stress (Wald *χ*²_1_=8.6; *q*=.01). For husbands, the results indicated a significant time×group interaction for symptoms of depression (Wald *χ*²_1_=13.2; *q*=.002). However, regarding anxiety, neither wives (Wald *χ*²_1_=1.8; *q*=.26) nor husbands (Wald *χ*²_1_=2.2; *q*=.24) had a significant time×group interaction. Nevertheless, in the intervention group, parental anxiety levels and perceived parental stress at T3 were significantly lower than in the control group (Multimedia Appendix 7).

#### Other Psychological Outcomes

Our findings also showed a significant time×group interaction for symptoms of sleep problems (Wald *χ*²_1_=6.0; *q*=.045), fatigue (Wald *χ*²_1_=5.7; *q*=.045), and perceived partner responsiveness (Wald *χ*²_1_=12.7; *q*=.003) among pregnant women at T2 (Multimedia Appendix 8). However, no such effect was observed for partners.

#### Mindfulness Level

Our findings showed a significant time×group interaction for mindfulness level at T2 among pregnant women (Wald *χ*²_1_=13.6; *P*<.001) and partners (Wald *χ*²_1_=4.7; *P*=.03) (Multimedia Appendix 9).

#### Infant Neuropsychological Development

The independent-samples *t* test results indicated that the average scores of the infants’ activity level (*t*_122_=3.845; *q* <.001), approach (*t*_122_=2.330, *q*=.049), intensity of reaction (*t*_122_=3.398; *q*=.004), quality of mood (*t*_122_=5.124; *q*<.001), distractibility (*t*_122_=4.087; *q*<.001), and adaptability (*t*_122_=3.138; *q*=.006) in the control group were statistically significantly higher than those in the intervention group, and the Cohen *d* values ranged from 0.42 to 0.92. However, no significant differences were found in the development of the 5 functional areas (communication, gross motor, fine motor, problem-solving, and personal-social) of infants (all *q*>.05) (Table 3).

**Table 3:** Differences in infant neuropsychological development between the intervention and control groups.

			Control group, mean (SD)		Intervention group, mean (SD)		2-tailed *t* (*df*)		*P* value		*q* value (FDR^a^ adjusted)		Cohen *d* (95% CI)
**Early Infancy Temperament Questionnaire**													
	Activity level	3.93 (0.84)		3.42 (0.63)		3.845 (122)		<.001		<.001		0.69 (0.33 to 1.06)	
	Rhythmicity	3.28 (0.51)		3.13 (0.59)		1.433 (122)		.16		.17		0.26 (–0.10 to 0.61)	
	Approach	3.16 (1.05)		2.75 (0.90)		2.330 (122)		.02		.049		0.42 (0.06 to 0.77)	
	Intensity of reaction	4.04 (1.02)		3.44 (0.96)		3.398 (122)		.001		.004		0.61 (0.25 to 0.97)	
	Attention span	3.48 (0.87)		3.33 (0.85)		0.969 (122)		.33		.27		0.17 (–0.17 to 0.53)	
	Distractibility	3.21 (0.90)		2.58 (0.81)		4.087 (122)		<.001		<.001		0.73 (0.37 to 1.10)	
	Threshold of reaction	4.05 (0.66)		4.08 (0.63)		–0.273 (122)		.79		.47		–0.05 (0 to 0.32)	
	Quality of mood	3.67 (0.68)		3.06 (0.65)		5.124 (122)		<.001		<.001		0.92 (0.55 to 1.29)	
	Adaptability	3.20 (1.13)		2.67 (0.71)		3.138 (122)		.002		.006		0.56 (0.20 to 0.92)	
**Age and Stages Questionnaire**–**Third Edition**													
	Communication	44.18 (11.63)		46.03 (10.28)		–0.94 0 (122)		.349		.43		–0.17 (0 to 0.51)	
	Gross motor	46.39 (12.72)		48.33 (13.14)		–0.835 (122)		.41		.44		–0.15 (0 to 0.49)	
	Fine motor	47.21 (9.81)		47.94 (9.66)		–0.414 (122)		.68		.44		–0.07 (0 to 0.41)	
	Problem-solving	50.16 (12.68)		52.38 (9.50)		–1.099 (122)		.27		.79		–0.20 (0 to 0.54)	
	Personal-social	39.75 (11.99)		43.57 (12.36)		–1.745 (122)		.08		.73		–0.31 (0 to 0.66)	

^a^FDR: false discovery rate.

#### Mediation Analysis

The 6 types of infants’ temperament scores (activity level, approach, adaptability, intensity of reaction, quality of mood, and distractibility) at 6 weeks in the intervention group were significantly lower than those in the control group. Therefore, we conducted a partial correlation analysis to explore the relationship between infants’ 6 types of temperament and parental psychological distress at T2, adjusting for psychological distress at T1.

The infants’ approach at T3 was positively related with maternal depressive symptoms (*r*=0.237; *P*=.01) and perceived stress (*r*=0.239; *P*=.01) at T2. Similarly, adaptability was positively associated with maternal depression (*r*=0.228; *P*=.01) and perceived stress (*r*=0.235; *P*=.01), intensity of reaction was positively associated with maternal depression (*r*=0.219; *P*=.02) and perceived stress (*r*=0.316; *P*=.001), quality of mood was positively associated with maternal depression symptoms (*r*=0.247; *P*=.007) and perceived stress (*r*=0.345; *P*<.001), and distractibility was positively correlated with maternal depression symptoms (*r*=0.254; *P*=.006) and perceived stress (*r*=0.318; *P*<.001). However, all 6 types of infant temperament were unrelated to paternal psychological distress at T2 (*P*>.05; Multimedia Appendix 10).

**Figure 4:**
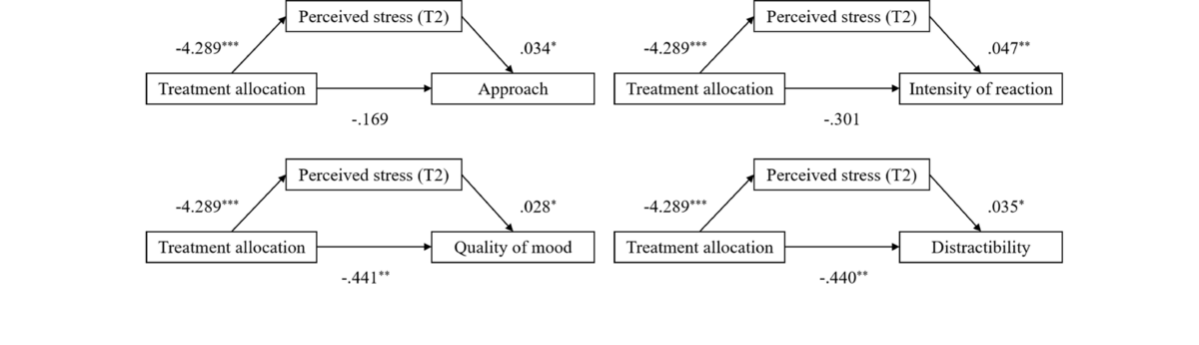
The mediating analysis of maternal psychological distress between treatment allocation and infant neuropsychological development. The path coefficients are unstandardized coefficients. Maternal perceived stress at T1 was adjusted. *P<.05; **P<.01;***P<.001; T1: baseline (12-20 weeks of gestation); T2: 2 weeks after completion of the intervention (approximately 20-28 weeks of gestation).

Based on the partial correlation results, we tested 10 mediation models, examining the indirect effect of treatment allocation (0=control group and 1=intervention group) on infant approach, intensity of reaction, quality of mood, distractibility, and adaptability through maternal depressive and perceived stress symptoms at T2. The mediating effect analysis showed that treatment allocation positively predicted maternal perceived stress at T2, and maternal perceived stress positively predicted infant approach, intensity of reaction, quality of mood, and distractibility (*P*<.05) (Multimedia Appendix 11). Bootstrap tests indicated that maternal perceived stress at T2 mediated the relationship between treatment allocation and infant approach (indirect effect=–0.148, 95% CI –0.324 to –0.006), intensity of reaction (indirect effect=–0.200, 95% CI –0.413 to –0.035), quality of mood (indirect effect=–0.122, 95% CI –0.250 to –0.020), and distractibility (indirect effect=–0.149, 95% CI –0.289 to –0.030), with all mediating effects reaching statistical significance (Figure 4).

## Discussion

### Primary Findings

Leveraging the convenience, accessibility, and flexibility of the WeChat mini-program, this study explored a moderately practical and effective approach to delivering psychological interventions for parental health. This holds particular significance in northwest China, where mental health resources are relatively scarce and economic and cultural development remains relatively constrained. Our findings indicated that couples who participated in the dMBI-EP experienced reduced psychological distress levels, as evidenced by lower average levels of depression and perceived stress compared with those in the control group post intervention. Moreover, the digital MBI appeared to have a ripple effect, contributing to infant temperament development at 6 weeks.

This study used a preventive intervention approach that achieved a small-to-medium effect on couples’ psychological distress and mindfulness levels. A systematic review and meta-analysis [17] published in 2023 indicated that the effect size of digital MBIs in reducing the psychological stress responses of pregnant women was small to medium. In terms of specific psychological metrics, the dMBI-EP most significantly affected depression in couples and more extensively affected pregnant women’s psychology, as it covered areas such as perceived stress, sleep, fatigue, and perceived partner responsiveness, in addition to depression, with less evident effects on these aspects in partners. However, this intervention had no significant impact on the parents’ general anxiety disorder symptoms. This differs from an earlier study on pregnant women with high anxiety symptoms (GAD-7≥10) [8]. Because our participants (especially husbands) had low baseline GAD-7 scores, we presume that our nonsignificant results may be associated with a floor effect, as there was little room for their anxiety to change.

Previous studies on couples’ interventions during pregnancy have yielded mixed results. In the Netherlands, a universal psychoeducational program for couples failed to reduce psychological distress or improve caregiving quality [67]. In contrast, a Korean couple-centered antenatal education program effectively decreased maternal prenatal depression and stress while enhancing parent–fetal attachment [68]. In the United Kingdom, a brief 4-week Mindfulness-Based Childbirth and Parenting program implemented within the National Health Service as a nonrandomized intervention demonstrated broader benefits; pregnant women showed reduced anxiety, depression, and pregnancy-related distress, whereas their partners exhibited decreased anxiety and depression, with potential stress reduction [69]. Another randomized intervention study from the United States found that the 4-week Mindful Transition to Parenthood Program significantly improved partners’ relationship satisfaction, mindfulness, and negative emotions with a medium-to-large effect size. However, for pregnant women, the program had no significant effect on any variable [15]. Systematic reviews and meta-analyses [17,18,70] have suggested that mindfulness interventions (including digital mindfulness interventions) during pregnancy may improve maternal prepartum and postpartum depression, anxiety, and stress, although their effects on husbands remain unclear. This study extends these findings by suggesting that mindfulness-based couple interventions could potentially produce both short- and long-term benefits for couples. However, the intervention had a greater effect on maternal psychological distress and other psychological outcomes (including sleep problems, fatigue, and perceived partner responsiveness) prepartum and postpartum, whereas its impact on paternal psychological distress was limited. One possible reason is that, in this study, husbands had milder baseline psychological stress responses than wives. However, even with relatively low baseline distress levels, husbands potentially benefited from the intervention. This may indicate the beneficial nature of our intervention and suggest the value of further improving and promoting it.

In addition, differences in the degree of participation in the interventions may account for this discrepancy. First, husbands may face a more acute shortage of time for the intervention than pregnant women. This can be attributed to the fact that husbands often face increased financial responsibilities and are generally less likely to receive work exemptions during pregnancy and the postpartum period. In contrast, some pregnant women may stop working for various reasons [71]. Based on the baseline results of this study, 78.8% of wives and 98.8% of husbands were employed. This is consistent with a study conducted in another province of China, which showed that the proportion of employed pregnant women was lower than that of their partners (72.2% vs 95.3%) [72]. Second, husbands often find it difficult to step into the role due to their inability to experience the same physical changes firsthand as pregnant women do [73], and the deep-rooted influence of traditional gender norms and stereotypes makes them perceive themselves often as playing a subsidiary role during pregnancy, viewing themselves primarily as providers, caregivers, protectors, and companions rather than central figures [74,75]. Some men believe that “pregnancy and childbirth are primarily the affairs of wives” or that “their presence in the experience was not acknowledged” and view themselves as “powerless,” “unimportant,” or “outsiders” [74,76]. Consequently, their motivation to participate in prenatal interventions is relatively low. Finally, the attractiveness of our intervention for husbands may be a concern. For a long time, husbands’ physical and psychological needs have seldom been addressed. This, in turn, has led to a widespread lack of understanding and targeted support [77]. Many existing prenatal health care service programs have also made fathers feel “alienated” [78]. This suggests that existing intervention measures should be improved based on further research on husbands (such as barriers to participating in the intervention and intervention preference) to align more closely with their concerns and needs.

### Secondary Findings

#### Duration of Learning and Practice and Outcome Improvement

The results of this study also revealed that the total number of class sessions on learning mindfulness and formal home practice was not significantly associated with parental psychological distress and mindfulness at T2. Research findings on the relationship between mindfulness practice frequency and intervention outcomes have been inconsistent. Some evidence shows a small yet significant association between practice and outcomes [79]. However, other evidence indicates that neither practice duration nor the frequency of practice days correlates with outcome improvements [80]. Our results support this hypothesis. The possible reasons for this irrelevance are as follows: first, the core factor contributing to the effectiveness of the intervention may not be the learning duration. Instead, elements such as intervention content and practice quality play a role [80], as everyone differs in learning input, comprehension, and the quality of their learning, all of which influence outcomes. Second, the distribution of the intervention content in terms of class hours was uneven, and there were differences in content density and course rhythm among the different course types and practice items. Third, informal mindfulness practice was not recorded because it might have been difficult to quantify and report accurately. However, compared to formal practice, informal mindfulness practice is brief and easy to implement. It represents an individualized way of developing a mindful state and integrating it into daily life [81,82], and has been proven to be more closely associated with improvements in intervention outcomes than formal practices [83,84]. Fourth, the recorded individual learning durations of some participants were based on the co-learning records of couples, which may have been inaccurate.

Interestingly, pregnant women who dropped out at T2 demonstrated higher baseline levels of mindfulness and maternal–fetal attachment. This could imply that these participants had already internalized basic mindfulness techniques and that the intervention might not have offered sufficient depth or challenges to keep them engaged. Future interventions should consider incorporating tiered or advanced content to maintain the interest and engagement of participants with varying levels of mindfulness experience.

#### The Ripple effect of dMBI-EP on Infant’s Neuropsychological Development

Our dMBI-EP seemingly exhibited a ripple effect on infant neuropsychological development at 6 weeks, influencing various dimensions such as activity, approach, intensity of reaction, quality of mood, distractibility, and adaptability, and presented a medium-to-large effect size. Moreover, the effects on the infants’ approach, intensity of reaction, quality of mood, and distractibility were mediated by reductions in maternal perceived stress symptoms at T2. These findings align with previous research indicating that maternal mindfulness during pregnancy can shape certain aspects of infant temperament, primarily through the reduction of maternal psychological distress [40,85]. Notably, given that both infants’ temperament and mothers’ psychological status can affect mother–infant interaction and bonding, which in turn influences mothers’ perception of their infants [86,87], and considering that our infant temperament results are solely based on mothers’ reports rather than objective measurements, it is difficult to distinguish the impact of confounding factors on our intervention results. However, the effect size of our intervention on infant temperament reached a medium-to-large magnitude. This suggests that the intervention likely had a tangible impact on the offspring despite potential reporting bias.

Our analysis revealed minimal evidence that MBIs improve infants’ neuropsychological development by alleviating the psychological distress of husbands. This finding may be attributable to several factors. First, the intervention demonstrated weaker effects in reducing psychological distress in husbands than in wives, potentially diminishing its downstream impact on infant outcomes. Second, given the fetus’s uterine development, paternal psychological states during pregnancy likely exert a limited direct physiological influence on fetal neurodevelopment. This mechanistic limitation is corroborated by observational studies showing consistent and robust associations between maternal prenatal distress and infant neuropsychological impairments [88], whereas investigations of paternal distress have yielded mixed findings. An American study showed that prenatal paternal stress predicts infant parasympathetic functioning at 7 months [89]. A birth cohort study in the Taiwan region of China found that prenatal paternal depression was associated with offspring tic disorders, developmental delays, developmental speech or language disorders, and developmental coordination disorder [90]. However, a United Kingdom study based on the Avon Longitudinal Study of Parents and Children population cohort found that paternal antenatal depression was not associated with an increased risk of anxiety [91]. A study in Norway found no evidence that paternal prenatal depressive symptoms could predict behavioral problems in children [92]. This study, which used an RCT design, revealed that reducing husbands’ prenatal psychological distress had a negligible impact on infants’ neurodevelopment, thus adding new evidence to this research field.

### Limitations

This study has several limitations. First, it was conducted at a single specialized hospital in Northwest China. The participants might have had relatively lower educational attainment and income level than those in Eastern China, which limits the generalizability of the findings to more diverse populations or regions with varying health care infrastructures. Second, this study relied on self-reports, which may have introduced subjective bias. Participants might have been influenced by social desirability, memory bias, or emotional states, potentially distorting the data. This could undermine the reliability of the results and warrants caution when interpreting our findings. Future work could assess the efficacy of dMBI-EP using diagnostic assessments and objective biological measurements for a more comprehensive clinical evaluation. Third, 38.0% (155/408) of the couples approached (one or both parties) declined to participate. As the mental health status of the participants who refused was not evaluated, we could not confirm the differences between those who refused and those who agreed to participate in this study’s population. This potential limitation may have affected the interpretation and generalizability of the findings. Fourth, although our original intention was to conduct a universal preventive intervention (ie, enroll all participants regardless of their baseline psychological distress status), only 12 (7.5%) couples had both parties testing negative for all 3 primary outcomes. Instead, this study ultimately included a high proportion of couples in which at least 1 partner had actual psychological distress and required timely intervention, achieving a natural enrichment of populations with a high need for psychological distress support. This outcome may be attributed to the high-stress recruitment setting for pregnant couples in the real world, coupled with self-selection bias among study participants, making it difficult for the sample to represent the general population and limiting the generalizability of the results. Fifth, intervention compliance was lower than ideal, especially among husbands. Such a shortcoming is relatively common in electronic interventions, particularly those that are “father-inclusive” [93]. Compliance regarding the “Coach Guiding” part was especially poor because participants had no unified available time and were generally not accustomed to joining strangers’ WeChat groups for relatively private discussions and sharing. This was highly unfavorable for promoting coaching in the form of WeChat group video conferences. Therefore, we had no choice but to provide guidance to participants through one-on-one online or face-to-face means; we also summarized the learning experiences of active sharers and disseminated them in the form of pictures and texts using WeChat.

### Conclusions

This RCT suggests that the dMBI-EP may have reduced psychological distress scores in couples, with potentially sustained benefits extending into the early postpartum period. More pronounced effects of the intervention were observed on maternal mental health, which might have mediated the positive influence of the intervention on multiple domains of infant neuropsychological development. Our findings tentatively contribute to the field in 3 ways. First, they provide empirical support for digital couple interventions as a promising approach to reducing perinatal mental health disparities, particularly in resource-limited settings. Second, they support and expand the “fetal programming” hypothesis by demonstrating maternal psychological states during pregnancy as modifiable factors that are likely to shape infant neuropsychological development. Finally, the findings highlight the significance of implementing interventions for couples and suggest the necessity of continuously and constructively exploring modes and methods of father-inclusive mental health interventions.

## Data Availability

The datasets are available from the corresponding author upon reasonable request.

## Appendix

Multimedia Appendix 1 CONSORT-eHEALTH checklist (V 1.6.1).

Multimedia Appendix 2 Mindfulness program outline.

Multimedia Appendix 3 Psychological Symptom Screening Positivity Among Expectant Parents.

Multimedia Appendix 4 Comparison of baseline information between dropout and non - dropout samples (T2).

Multimedia Appendix 5 Comparison of baseline information between dropout and non - dropout samples(T3).

Multimedia Appendix 6 Linear regression analyses of total class sessions for learning mindfulness and formal home practice on parental psychological stress response outcomes.

Multimedia Appendix 7 Overall test results and between - group differences in postpartum parental psychological distress in the generalized estimating equations analysis.

Multimedia Appendix 8 Overall test results and between - group differences in other psychological outcomes of expectant parents in the generalized estimating equations analysis.

Multimedia Appendix 9 Overall test results and between-group differences in expectant parental mindfulness level in the generalized estimating equations analysis.

Multimedia Appendix 10 Correlation between expectant parental psychological distress during pregnancy and infant temperament.

Multimedia Appendix 11 The mediating role of maternal perceived stress (T2) between treatment allocation and infant temperament.

## Abbreviations

AIS: Athens Insomnia Scale
CONSORT-EHEALTH: Consolidated Standards of Reporting Trials of Electronic and Mobile Health Interventions and Online Telehealth
dMBI-EP: digital mindfulness-based intervention for expectant parents
EPDS: Edinburgh Postpartum Depression Scale
GAD-7: Generalized Anxiety Disorder Questionnaire
GEE: generalized estimated equation
MAAS: Maternal Antenatal Attachment Scale
MBI: mindfulness-based intervention
PAAS: Paternal Antenatal Attachment Scale
PSS-10: Perceived Stress Scale
RCT: randomized controlled trial
