# Supplementary material for: Effects of Digital Mindfulness Training for Couples on Psychological Distress and Infant Neuropsychological Development: Randomized Controlled Trial
Source: J Med Internet Res. 2025 Nov 21;27:e77260. doi: 10.2196/77260 (PMC12680938; doi:10.2196/77260)
Supplement: Multimedia Appendix 2 [file jmir_v27i1e77260_app2.docx]

**Multimedia Appendix 2**. Mindfulness program outline.

| **Module**  **(week)** | **Theme** | **Content** | **Home practice** | |
| --- | --- | --- | --- | --- |
|  |  |  | **Formal practice**  (With audio guidance) | **Informal practice** |
| 1 | Introduction and approach to Mindfulness | 1.1 Prenatal psychological distress of expectant parents: causes, manifestations and negative effects.  1.2 Overview of all course: introduction to mindfulness, purposes and effects of mindfulness intervention, weekly course themes, importance of mindfulness practice.  1.3 Demonstration: mindful raisin eating. | Body scan | Mindful Eating |
| 2 | Be aware of the present experience and wake up from auto-navigation mode | 1.1 Introduction to the auto-navigation mode.  1.2 Be aware of the present experience; be conscious of the concentration, wandering and retrieval of attention.  1.3 Identify and break away from the auto-navigation mode. | Mindful breathing | Mindfulness in daily life (Mindful brushing, mindful bathing, etc.) |
| The end of 2nd week | Coach Guiding  Q&A, encouragement and sharing | 1.1 Understand the learning experience of expectant parents and clear their doubts.  1.2 Encourage expectant parents to keep practicing, practice together and share experiences of mindfulness.  1.3 Guide the communication and sharing of mindfulness experiences among couples. | - | - |
| 3 | Identify avoidance reactions, learn to accept, and coexist with difficulties | 1.1 Identify avoidance reactions of expectant parents during pregnancy.  1.2 Allow everything to be as it is and learn to accept.  1.3 Be aware of unpleasant experiences in pregnancy and practice coexisting with them.  1.4 Demonstration: 3 - minute breath space exercise. | Mindful sitting meditation | 3 - minute Breath Space Exercise |
| 4 | Mom, Dad and the baby together | 1.1 Introduce the fetus' perceptual and sensory development.  1.2 Explain how to interact with the fetus during pregnancy, such as mindful touching of fetal movement, mindful listening to the fetal heartbeat with the help of a fetal heart monitor. | Body scan | Mindfully touch the fetal movement and mindfully listen to the fetal heartbeat (Utilize a fetal heart monitor) |
| 5 | Preparing for childbirth | 1.1 Explain the physiology of normal childbirth.  1.2 Explain mindful stretching to prepare the body for childbirth.  1.3 Introduce mindfulness in dealing with the pain of childbirth.  1.4 Demonstration: mindful stretching. | Mindful stretching, Ice-holding exercise | 3 - minute Breath Space Exercise |
| 6 | Mindfulness nourishes a happy life | 1.1 Learn mindful eye contact, hugging, listening/speaking to support each other.  1.2 Summarize the key points of mindfulness practice during pregnancy.  1.3 Emphasize the necessity of continuous mindfulness practice. | Choose one from the previous exercises for practice | Daily Mindful Communication |
| The end of 6nd week | Coach Guiding  Q&A, encouragement, sharing and summary | 1.1 Understand the learning experience of expectant parents and clear their doubts.  1.2 Guide the communication and sharing of mindfulness practice experiences among different families.  1.3 Encourage expectant parents to continue practicing mindfulness together.  1.4 Make a summary of the course. | - | - |
