# Supplementary material for: Effects of Digital Mindfulness Training for Couples on Psychological Distress and Infant Neuropsychological Development: Randomized Controlled Trial
Source: J Med Internet Res. 2025 Nov 21;27:e77260. doi: 10.2196/77260 (PMC12680938; doi:10.2196/77260)
Supplement: Multimedia Appendix 3 [file jmir_v27i1e77260_app3.docx]

**Multimedia Appendix 3.** Psychological Symptom Screening Positivity Among Expectant Parents (N=160 couples).

|  | **Expectant Mothers n (%)** | **Expectant Fathers**  **n (%)** | **Both parents**  **n (%)** | **Only one parent**  **n (%)** | **Neither parent**  **n (%)** |
| --- | --- | --- | --- | --- | --- |
| EPDS≥9 | 98 (61.3) | 47 (29.4) | 31(19.4) | 83 (51.9) | 46 (28.8) |
| GAD-7≥5 | 90 (56.3) | 51 (31.9) | 35 (21.9) | 71 (44.4) | 54 (33.8) |
| PSS-10≥14 | 96 (60.0) | 85 (53.1) | 58 (36.3) | 65 (40.6) | 37 (23.1) |
| EPDS≥9, GAD-7≥5,  or PSS-10≥14 (any) | 134 (83.8) | 106 (66.3) | 92 (57.5) | 56 (35.0) | 12 (7.5) |
| EPDS≥9, GAD-7≥5, and PSS-10≥14 (all) | 57 (35.6) | 23 (14.4) | 11 (6.9) | 58 (36.3) | 91 (56.9) |

EPDS: Edinburgh Postnatal Depression Scale; GAD-7: Generalized Anxiety Disorder Scale; PSS-10: Perceived Stress Scale.
