# Supplementary material for: Effects of Digital Mindfulness Training for Couples on Psychological Distress and Infant Neuropsychological Development: Randomized Controlled Trial
Source: J Med Internet Res. 2025 Nov 21;27:e77260. doi: 10.2196/77260 (PMC12680938; doi:10.2196/77260)
Supplement: Multimedia Appendix 4 [file jmir_v27i1e77260_app4.docx]

**Multimedia Appendix 4** Comparison of baseline information between dropout and non - dropout samples (T2)

|  | |  | **Non - dropout** | | | | |  | **Dropout** | ***P*** |
| --- | --- | --- | --- | --- | --- | --- | --- | --- | --- | --- |
|  | |  | **mean±SD/n (%)** | | | | |  | **mean±SD/n (%)** |  |
| **Number of Individuals** | | |  | | | | |  |  |  |
| **Expectant Mother** | | |  | | | | |  |  | 0.276 |
|  | | Intervention group | 78 (97.5) | | | | |  | 2 (2.5) |  |
|  | | Control group | 74 (92.5) | | | | |  | 6 (7.5) |  |
| **Expectant Father** | | |  | | | | |  |  | 0.564 |
|  | | Intervention group | 75 (93.8) | | | | |  | 5 (6.3) |  |
|  | | Control group | 72 (90.0) | | | | |  | 8 (10.0) |  |
| **General Baseline Demographics** | | |  | | | | |  |  |  |
| **Expectant Mother** | | |  | | | | |  |  |  |
|  | | **Age (years)** | 26.87±2.81 | | | | |  | 27.38±2.33 | 0.617 |
|  | | **Nationality** |  | | | | |  |  | 0.803 |
|  | | Han | 128 (94.8) | | | | |  | 7 (5.2) |  |
|  | | National minority | 24 (96.0) | | | | |  | 1 (4.0) |  |
|  | | **Education** |  | | | | |  |  | 0.402 |
|  | | High school or less | 24 (92.3) | | | | |  | 2 (7.7) |  |
|  | | Junior college | 53 (93.0) | | | | |  | 4 (7.0) |  |
|  | | Undergraduate or above | 75 (97.4) | | | | |  | 2 (2.6) |  |
|  | | **Gestational age (weeks)** | 14.13±2.58 | | | | |  | 13.75±2.19 | 0.687 |
|  | | **Adverse pregnancy history** | |  | | | |  |  | 0.645 |
|  | | Yes | 28 (93.3) | | | | |  | 2 (6.7) |  |
|  | | No | 124 (95.4) | | | | |  | 6 (4.6) |  |
|  | | **Complications during pregnancy** | | | |  | |  |  | 0.388 |
|  | | Yes | 34 (91.9) | | | | |  | 3 (8.1) |  |
|  | | No | 118 (95.9) | | | | |  | 5 (4.1) |  |
|  | | **Current employment status** |  | | | | |  |  | 1.000 |
|  | | Yes | 119 (94.4) | | | | |  | 7 (5.6) |  |
|  | | No | 33 (97.1) | | | | |  | 1 (2.9) |  |
|  | | **Pre-pregnancy BMI (kg/m^2^)** | 21.80±3.45 | | | | |  | 21.29±2.74 | 0.684 |
|  | | **Current BMI (kg/m^2^)** | 22.42±3.55 | | | | |  | 22.12±2.40 | 0.813 |
|  | | **Residence** |  | | | | |  |  | 0.637 |
|  | | Urban | 125 (95.4) | | | | |  | 6 (4.6) |  |
|  | | Rural | 27 (93.1) | | | | |  | 2 (6.9) |  |
|  | | **Monthly household income (Yuan)** | | | | | |  |  | 0.394 |
|  | | <5000 | 30 (96.8) | | | | |  | 1 (3.2) |  |
|  | | 5000~8999 | 80 (96.4) | | | | |  | 3 (3.6) |  |
|  | | ≥9000 | 42 (91.3) | | | | |  | 4 (8.7) |  |
|  | | **Both spouses intended this pregnancy** | | | | | |  |  | 1.000 |
|  | | Yes | 104 (94.5) | | | | |  | 6 (5.5) |  |
|  | | No | 48 (96.0) | | | | |  | 2 (4.0) |  |
|  | | **Mode of pregnancy** |  | | | | |  |  | 1.000 |
|  | | Natural pregnancy | 151 (95.0) | | | | |  | 8 (5.0) |  |
|  | | Assisted pregnancy | 1 (100.0) | | | | |  | 0 (0.0) |  |
| **Expectant Father** | | |  | | | | |  |  |  |
|  | | **Age (years)** | 29.15±3.26 | | | | |  | 27.46±2.07 | 0.069 |
|  | | **Nationality** |  | | | | |  |  | 0.694 |
|  | | Han | 123 (91.1) | | | | |  | 12 (8.9) |  |
|  | | National minority | 24 (96.0) | | | | |  | 1 (4.0) |  |
|  | | **Education** |  | | | | |  |  | 0.092 |
|  | | High school or less | 28 (84.8) | | | | |  | 5 (15.2) |  |
|  | | Junior college | 54 (94.7) | | | | |  | 3 (5.3) |  |
|  | | Undergraduate or above | 65 (92.9) | | | | |  | 5 (7.1) |  |
|  | | **Current Employment Status** |  | | | | |  |  | 1.000 |
|  | | Yes | 145 (91.8) | | | | |  | 13 (8.2) |  |
|  | | No | 2 (100.0) | | | | |  | 0 (0.0) |  |
|  | | **Current BMI (kg/m^2^)** | 24.19±3.58 | | | | |  | 24.24±2.55 | 0.957 |
|  | | **Wives’** **current employment status** |  | | | | |  |  | 0.072 |
|  | | Yes | 113 (89.7) | | | | |  | 13 (10.3) |  |
|  | | No | 34 (100.0) | | | | |  | 0 (0.0) |  |
|  | | **Residence** |  | | | | |  |  | 0.256 |
|  | | Urban | 122 (93.1) | | | | |  | 9 (6.9) |  |
|  | | Rural | 25 (86.2) | | | | |  | 4 (13.8) |  |
|  | | **Monthly household income (Yuan)** |  | |  | | | | | 0.307 |
|  | | <5000 | 30 (96.8) | | | | |  | 1 (3.2) |  |
|  | | 5000~8999 | 74 (89.2) | | | | |  | 9 (10.8) |  |
|  | | ≥9000 | 43 (93.5) | | | | |  | 3 (6.5) |  |
|  | | **Both spouses intended this pregnancy** |  | | | | | | | 0.229 |
|  | | Yes | 103 (93.6) | | | | |  | 7 (6.4) |  |
|  | | No | 44 (88.0) | | | | |  | 6 (12.0) |  |
|  | | **Wives’ gestational age (Weeks)** | 14.11±2.57 | | | | |  | 14.08±2.50 | 0.966 |
|  | | **Mode of pregnancy** |  | | | | |  |  | 1.000 |
|  | | Natural pregnancy | 146 (91.8) | | | | |  | 13 (8.2) |  |
|  | | Assisted pregnancy | 1 (100.0) | | | | |  | 0 (0.0) |  |
| **Primary Outcome Indicators** | | |  | | | | |  |  |  |
|  | | Maternal depression | 9.56±5.17 | | | | |  | 11.50±3.59 | 0.297 |
|  | | Paternal depression | 6.63±5.06 | | | | |  | 5.38±3.36 | 0.385 |
|  | | Maternal anxiety | 5.44±3.61 | | | | |  | 5.37±2.67 | 0.960 |
|  | | Paternal anxiety | 3.25±3.18 | | | | |  | 4.15±2.85 | 0.324 |
|  | | Maternal perceived stress | 14.90±5.38 | | | | |  | 14.50±1.60 | 0.582 |
|  | | Paternal perceived stress | 13.21±5.70 | | | | |  | 13.15±5.38 | 0.972 |
| **Secondary Outcome Indicators** | | |  | | | | |  |  |  |
|  | | Maternal sleep problems | 5.57±3.35 | | | | |  | 4.75±2.87 | 0.497 |
|  | | Paternal sleep problems | 3.88±3.20 | | | | |  | 3.46±2.47 | 0.644 |
|  | | Maternal fatigue | 6.56±3.12 | | | | |  | 5.00±2.39 | 0.166 |
|  | | Paternal fatigue | 4.24±2.97 | | | | |  | 4.62±2.18 | 0.656 |
| Maternal perceived partner responsiveness | | | | | | 5.17±1.15 |  | 5.52±1.51 | 0.413 |  |
| Paternal perceived partner responsiveness | | | | | | 5.39±1.06 |  | 5.60±0.80 | 0.489 |  |
|  | | Maternal antenatal attachment | 69.43±8.13 | | | | |  | 75.88±3.80 | 0.028 |
|  | | Paternal antenatal attachment | 59.32±6.06 | | | | |  | 56.23±9.16 | 0.254 |
| **Operational Variable** | | |  | | | | |  |  |  |
|  | | Maternal mindfulness | 62.20±7.48 | | | | |  | 68.38±11.98 | 0.029 |
|  | | Paternal mindfulness | 62.07±9.03 | | | | |  | 63.77±11.50 | 0.526 |
