# Supplementary material for: Effects of Digital Mindfulness Training for Couples on Psychological Distress and Infant Neuropsychological Development: Randomized Controlled Trial
Source: J Med Internet Res. 2025 Nov 21;27:e77260. doi: 10.2196/77260 (PMC12680938; doi:10.2196/77260)
Supplement: Multimedia Appendix 5 [file jmir_v27i1e77260_app5.docx]

**Multimedia Appendix 5** Comparison of baseline information between dropout and non - dropout samples(T3).

|  |  | | **Non - dropout** | | | | |  | **Dropout** | ***P*** |
| --- | --- | --- | --- | --- | --- | --- | --- | --- | --- | --- |
|  |  | | **mean±SD / n (%)** | | | | |  | **mean±SD /n (%)** |  |
| **Number of Individuals** | | |  | | | | |  |  |  |
| **Expectant Mother** | | |  | | | | |  |  | 0.365 |
|  | Intervention group | | 62(77.5) | | | | |  | 18(22.5) |  |
|  | Control group | | 57(71.3) | | | | |  | 23(28.8) |  |
| **Expectant Father** | | |  | | | | |  |  | 0.574 |
|  | Intervention group | | 63(78.8) | | | | |  | 17(21.3) |  |
|  | Control group | | 60(75.0) | | | | |  | 20(25.0) |  |
| **General Baseline Demographics** | | |  | | | | |  |  |  |
| **Expectant Mother** | | |  | | | | |  |  |  |
|  | **Age (years)** | | 26.87±2.65 | | | | |  | 26.98±3.17 | 0.828 |
|  | **Nationality** | |  | | | | |  |  | 0.427 |
|  | Han | | 102(75.6) | | | | |  | 33(24.4) |  |
|  | National minority | | 17(68.0) | | | | |  | 8(32.0) |  |
|  | **Education** | |  | | | | |  |  | 0.748 |
|  | High school or less | | 18(69.2) | | | | |  | 8(30.8) |  |
|  | Junior college | | 42(73.7) | | | | |  | 15(26.3) |  |
|  | Undergraduate or above | | 59(76.6) | | | | |  | 18(23.4) |  |
|  | **Gestational age (weeks)** | | 14.04±2.52 | | | | |  | 14.29±2.68 | 0.589 |
|  | **Adverse pregnancy history** | | | |  | | |  |  | 0.750 |
|  | Yes | | 23(76.7) | | | | |  | 7(23.3) |  |
|  | No | | 96(73.8) | | | | |  | 34(26.2) |  |
|  | **Complications during pregnancy** | | |  | | | |  |  | 0.824 |
|  | Yes | | 27(73.0) | | | | |  | 10(27.0) |  |
|  | No | | 92(74.8) | | | | |  | 31(25.2) |  |
|  | **Current employment status** | |  | | | | |  |  | 0.752 |
|  | Yes | | 93(73.8) | | | | |  | 33(26.2) |  |
|  | No | | 26(76.5) | | | | |  | 8(23.5) |  |
|  | **Pre-pregnancy BMI (kg/m^2^)** | | 21.70±3.51 | | | | |  | 21.97±3.15 | 0.669 |
|  | **Current BMI (kg/m^2^) (kg/m^2^)** | | 22.36±3.66 | | | | |  | 22.52±2.98 | 0.797 |
|  | **Residence** | |  | | | | |  |  | 0.461 |
|  | Urban | | 99(75.6) | | | | |  | 32(24.4) |  |
|  | Rural | | 20(69.0) | | | | |  | 9(31.0) |  |
|  | **Monthly household income (Yuan)** | | | | | | |  |  | 0.166 |
|  | <5000 | | 26(83.9) | | | | |  | 5(16.1) |  |
|  | 5000~8999 | | 63(75.9) | | | | |  | 20(24.1) |  |
|  | ≥9000 | | 30(65.2) | | | | |  | 16(34.8) |  |
|  | **Both spouses intended this pregnancy** | | | | | | |  |  | 0.942 |
|  | Yes | | 82(74.5) | | | | |  | 28(25.5) |  |
|  | No | | 37(74.0) | | | | |  | 13(26.0) |  |
|  | **Mode of pregnancy** | |  | | | | |  |  | 1.000 |
|  | Natural pregnancy | | 118(74.2) | | | | |  | 41(25.8) |  |
|  | Assisted pregnancy | | 1(100.0) | | | | |  | 0(0.0) |  |
| **Expectant Father** | | |  | | | | |  |  |  |
|  | **Age (Years)** | | 29.07±3.25 | | | | |  | 28.84±3.09 | 0.707 |
|  | **Nationality** | |  | | | | |  |  | 0.069 |
|  | Han | | 100(74.1) | | | | |  | 35(25.9) |  |
|  | National minority | | 23(92.0) | | | | |  | 2(8.0) |  |
|  | **Education** | |  | | | | |  |  | 0.693 |
|  | High school or less | | 25(75.8) | | | | |  | 8(24.2) |  |
|  | Junior college | | 42(73.7) | | | | |  | 15(26.3) |  |
|  | Undergraduate or above | | 56(80.0) | | | | |  | 14(20.0) |  |
|  | **Current employment status** | |  | | | | |  |  | 0.435 |
|  | Yes | | 121(76.6) | | | | |  | 37(23.4) |  |
|  | No | | 2(100.0) | | | | |  | 0(0.0) |  |
|  | **Wives’ current employment status** | | | | | |  |  |  | 0.693 |
|  | Yes | | 96(76.2) | | | | |  | 30(23.8) |  |
|  | No | | 27(79.4) | | | | |  | 7 (20.6) |  |
|  | **Current BMI (kg/m^2^)** | | 23.93±3.48 | | | | |  | 25.05±3.50 | 0.087 |
|  | **Residence** | |  | | | | |  |  | 0.886 |
|  | Urban | | 101(77.1) | | | | |  | 30(22.9) |  |
|  | Rural | | 22(75.9) | | | | |  | 7(24.1) |  |
|  | **Monthly household income (Yuan)** | | | | | | |  |  | 0.173 |
|  | <5000 | | 26(83.9) | | | | |  | 5(16.1) |  |
|  | 5000~8999 | | 66(79.5) | | | | |  | 17(20.5) |  |
|  | ≥9000 | | 31(67.4) | | | | |  | 15(32.6) |  |
|  | **Both spouses intended this pregnancy** |  | | | | | | | | 0.527 |
|  | Yes | | 83(75.5) | | | | |  | 27(24.5) |  |
|  | No | | 40(80.0) | | | | |  | 10(20.0) |  |
|  | **Wives’ gestational age (Weeks)** | 14.09±2.52 | | | | | |  | 14.16±2.71 | 0.880 |
|  | **Mode of pregnancy** | |  | | | | |  |  | 1.000 |
|  | Natural pregnancy | | 122(76.7) | | | | |  | 37(23.3) |  |
|  | Assisted pregnancy | | 1(100.0) | | | | |  | 0(0.0) |  |
| **Primary Outcome Indicators** | | |  | | | | |  |  |  |
|  | Maternal depression | | 9.69±5.40 | | | | |  | 9.56±4.25 | 0.891 |
|  | Paternal depression | | 6.62±5.00 | | | | |  | 6.24±4.83 | 0.688 |
|  | Maternal anxiety | | 5.41±3.71 | | | | |  | 5.51±3.13 | 0.877 |
|  | Paternal anxiety | | 3.36±3.36 | | | | |  | 3.22±2.42 | 0.812 |
|  | Maternal perceived stress | | 15.23±5.02 | | | | |  | 13.88±5.85 | 0.157 |
|  | Paternal perceived stress | | 13.30±5.77 | | | | |  | 12.92±5.33 | 0.726 |
| **Secondary Outcome Indicators** | | |  | | | | |  |  |  |
|  | Maternal sleep problems | | 5.42±3.34 | | | | |  | 5.85±3.30 | 0.473 |
|  | Paternal sleep problems | | 3.92±3.28 | | | | |  | 3.62±2.68 | 0.616 |
|  | Maternal fatigue | | 6.59±3.03 | | | | |  | 6.17±3.29 | 0.458 |
|  | Paternal fatigue | | 4.27±3.42 | | | | |  | 4.55±3.47 | 0.687 |
| Maternal perceived partner responsiveness | | | | | | 5.44±0.96 | |  | 5.34±1.27 | 0.345 |
| Paternal perceived partner responsiveness | | | | | | 5.38±1.23 | |  | 5.38±1.23 | 0.843 |
|  | Maternal antenatal attachment | | 70.11±8.13 | | | | |  | 68.68±7.98 | 0.329 |
|  | Paternal antenatal attachment | | 59.30±6.75 | | | | |  | 58.30±4.99 | 0.404 |
| **Operational Variable** | | |  | | | | |  |  |  |
|  | Maternal mindfulness | | 62.03±6.76 | | | | |  | 63.90±10.29 | 0.281 |
|  | Paternal mindfulness | | 62.47±9.30 | | | | |  | 61.32±9.06 | 0.509 |
