# Supplementary material for: Effects of Digital Mindfulness Training for Couples on Psychological Distress and Infant Neuropsychological Development: Randomized Controlled Trial
Source: J Med Internet Res. 2025 Nov 21;27:e77260. doi: 10.2196/77260 (PMC12680938; doi:10.2196/77260)
Supplement: Multimedia Appendix 6 [file jmir_v27i1e77260_app6.docx]

**Multimedia Appendix 6.** Linear regression analyses of total class sessions for learning mindfulness and formal home practice on parental psychological stress response outcomes.

| **Outcomes（T2）** | ***B*** | ***SE*** | ***β*** | ***t*** | ***P*** | ***95%CI*** | | ***R^2^*** | **adjusted *R^2^*** | ***ΔR²*** |
| --- | --- | --- | --- | --- | --- | --- | --- | --- | --- | --- |
| Maternal depression | -0.096 | 0.051 | -0.255 | -1.904 | 0.062 | -0.198 | 0.005 | 0.341 | 0.126 | 0.030 |
| Maternal anxiety | -0.046 | 0.029 | -0.188 | -1.565 | 0.123 | -0.105 | 0.013 | 0.444 | 0.261 | 0.067 |
| Maternal perceived stress | -0.092 | 0.056 | -0.195 | -1.628 | 0.109 | -0.205 | 0.021 | 0.443 | 0.261 | 0.023 |
| Paternal depression | 0.005 | 0.058 | 0.011 | 0.079 | 0.937 | -0.112 | 0.121 | 0.210 | -0.044 | 0.060 |
| Paternal anxiety | 0.016 | 0.040 | 0.050 | 0.389 | 0.699 | -0.065 | 0.096 | 0.242 | -0.001 | 0.082 |
| Paternal perceived stress | -0.054 | 0.094 | -0.075 | -0.570 | 0.571 | -0.242 | 0.135 | 0.288 | 0.059 | 0.019 |
| Maternal mindfulness | 0.162 | 0.095 | 0.211 | 1.711 | 0.092 | -0.028 | 0.352 | 0.414 | 0.222 | 0.092 |
| Paternal mindfulness | 0.090 | 0.125 | 0.091 | 0.718 | 0.476 | -0.161 | 0.341 | 0.367 | 0.163 | 0.062 |

Note: Demographic variables were controlled for both expectant parents. These included residence, average monthly household income, age, ethnicity, highest educational level, employment status, current BMI, gestational weeks, pregnancy method, history of adverse pregnancy and childbirth, presence of complications, mode of pregnancy, and whether the pregnancy was planned by both parties. And the corresponding baseline levels of psychological stress reaction symptoms were also controlled. In addition, the age, ethnicity, employment status, and highest educational level of their spouses were controlled. Moreover, for expectant mothers, pre - pregnancy BMI was controlled.
