# Supplementary material for: Effects of Digital Mindfulness Training for Couples on Psychological Distress and Infant Neuropsychological Development: Randomized Controlled Trial
Source: J Med Internet Res. 2025 Nov 21;27:e77260. doi: 10.2196/77260 (PMC12680938; doi:10.2196/77260)
Supplement: Multimedia Appendix 7 [file jmir_v27i1e77260_app7.docx]

**Multimedia Appendix 7.** Overall test results and between - group differences in postpartum parental psychological distress in the generalized estimating equations analysis.

| **Outcomes and time** | **Control group, (mean±SD)** | **Intervention group, (mean±SD)** | **Estimated mean difference,**  **mean (95% CI)** | ***Cohen's d*** | ***Group × time*** | | |
| --- | --- | --- | --- | --- | --- | --- | --- |
|  |  |  |  |  | ***Waldχ^2^ (df)*** | ***P*** | **FDR**  ***q* value** |
| **Maternal depression** |  |  |  |  |  |  |  |
| T1 | 10.04±4.71 | 9.28±5.50 | -0.76 (-2.34, 0.81) |  |  |  |  |
| T3 | 11.33±6.42 | 7.06±5.70 | -4.90 (-6.96, -2.84) | 0.66 (0.31, 1.00) | 13.541 | <0.001 | 0.002 |
| **Paternal depression** |  |  |  |  |  |  |  |
| T1 | 6.13±4.38 | 6.94±5.45 | 0.81 (-0.71, 2.34) |  | 13.189 | <0.001 | 0.002 |
| T3 | 7.33±4.53 | 5.22±4.72 | -2.49 (-4.10, -0.88) | 0.46 (0.10, 0.81) |  |  |  |
| **Maternal anxiety** |  |  |  |  |  |  |  |
| T1 | 5.84±3.70 | 5.04±3.39 | -0.80 (-1.89, 0.29) |  | 1.788 | 0.181 | 0.259 |
| T3 | 6.60±4.12 | 4.94±3.59 | -1.83 (-3.18, -0.48) | 0.40 (0.06, 0.74) |  |  |  |
| **Paternal anxiety** |  |  |  |  |  |  |  |
| T1 | 3.65±3.09 | 3.00±3.20 | -0.65 (-1.62, 0.32) |  | 2.223 | 0.136 | 0.238 |
| T3 | 4.93±3.61 | 3.63±3.58 | -1.59 (-2.81, -0.38) | 0.34 (0.004, 0.68) |  |  |  |
| **Maternal perceived stress** | |  |  |  |  |  |  |
| T1 | 14.91±5.08 | 14.85±5.46 | -0.06 (-1.69, 1.56) |  | 8.561 | 0.003 | 0.014 |
| T3 | 17.16±5.30 | 13.73±8.16 | -3.60 (-5.99, -1.20) | 0.47 (0.13, 0.81) |  |  |  |
| **Paternal perceived stress** | |  |  |  |  |  |  |
| T1 | 12.95±5.34 | 13.46±5.98 | 0.51 (-1.23, 2.26) |  | 7.573 | 0.006 | 0.021 |
| T3 | 13.67±6.77 | 11.17±7.30 | -2.75 (-5.13, -0.38) | 0.33 (-0.003, 0.67) |  |  |  |

T1: baseline (12 to 20 weeks of gestation); T3: six weeks postpartum.
