# Supplementary material for: Effects of Digital Mindfulness Training for Couples on Psychological Distress and Infant Neuropsychological Development: Randomized Controlled Trial
Source: J Med Internet Res. 2025 Nov 21;27:e77260. doi: 10.2196/77260 (PMC12680938; doi:10.2196/77260)
Supplement: Multimedia Appendix 8 [file jmir_v27i1e77260_app8.docx]

**Multimedia Appendix 8.** Overall test results and between - group differences in other psychological outcomes of expectant parents in the generalized estimating equations analysis.

| **Outcomes** | **Control group, (mean±SD)** | **Intervention group, (mean±SD)** | **Estimated mean difference,**  **mean (95% CI)** | ***Cohen’d*** | ***Group × time*** | | | |
| --- | --- | --- | --- | --- | --- | --- | --- | --- |
|  |  |  |  |  | ***Waldχ^2^ (df)*** | ***P*** value | **FDR**  *q* value | |
| **Maternal sleep problems** | |  |  |  |  |  | |  |
| T1 | 5.63±3.49 | 5.44±3.17 | -0.19 (-1.21, 0.84) |  | 6.020 | 0.014 | | 0.045 |
| T2 | 6.26±3.08 | 4.67±2.68 | -1.60 (-2.51, -0.69) | 0.55 (0.23, 0.88) |  |  | |  |
| **Paternal sleep problems** | |  |  |  |  |  | |  |
| T1 | 4.03±3.09 | 3.68±3.21 | -0.35 (-1.32, 0.62) |  | 0.098 | 0.754 | | 0.754 |
| T2 | 4.36±3.08 | 3.80±2.95 | -0.51 (-1.46, 0.45) | 0.19 (-0.14, 0.51) |  |  | |  |
| **Maternal fatigue** | |  |  |  |  |  | |  |
| T1 | 6.55±2.81 | 6.41±3.37 | -0.14 (-1.09, 0.82) |  | 5.710 | 0.017 | | 0.045 |
| T2 | 6.91±3.21 | 5.49±3.27 | -1.39 (-2.41, -0.38) | 0.44 (0.11, 0.76) |  |  | |  |
| **Paternal fatigue** | |  |  |  |  |  | |  |
| T1 | 4.65±3.09 | 3.89±2.69 | -0.76 (-1.65, 0.13) |  | 0.118 | 0.732 | | 0.754 |
| T2 | 4.63±3.58 | 4.05±3.27 | -0.58 (-1.67, 0.50) | 0.17 (-0.16, 0.49) |  |  | |  |
| **Maternal perceived partner responsiveness** | | |  |  |  |  | |  |
| T1 | 5.27±1.16 | 5.11±1.18 | -0.15 (-0.51, 0.21) |  | 12.654 | <0.001 | | 0.003 |
| T2 | 4.93±1.18 | 5.44±0.98 | 0.49 (0.15, 0.84) | -0.47 (0.17, 0.79) |  |  | |  |
| **Paternal perceived partner responsiveness** | | |  |  |  |  | |  |
| T1 | 5.47±1.00 | 5.36±1.09 | -0.11 (-0.43, 0.21) |  | 0.824 | 0.364 | | 0.485 |
| T2 | 5.17±1.32 | 5.26±1.23 | 0.09 (-0.32, 0.49) | -0.07 (0, 0.37) |  |  | |  |
| **Maternal antenatal attachment** | |  |  |  |  |  | |  |
| T1 | 69.45±7.75 | 70.05±8.46 | 0.60 (-1.90, 3.10) |  | 3.086 | 0.079 | | 0.158 |
| T2 | 70.30±7.94 | 73.21±7.62 | 2.73 (0.14, 5.04) | -0.37 (0.07, 0.69) |  |  | |  |
| **Paternal antenatal attachment** | |  |  |  |  |  | |  |
| T1 | 59.19±5.98 | 58.95±6.81 | -0.24 (-2.21, 1.74) |  | 1.098 | 0.295 | | 0.472 |
| T2 | 59.81±5.90 | 60.67±6.12 | 0.79 (-1.14, 2.72) | -0.14 (0, 0.46) |  |  | |  |

T1: baseline (12 to 20 weeks of gestation); T2: two weeks after the completion of intervention (approximately 20 to 28 weeks of gestation).
