# Supplementary material for: Effects of Digital Mindfulness Training for Couples on Psychological Distress and Infant Neuropsychological Development: Randomized Controlled Trial
Source: J Med Internet Res. 2025 Nov 21;27:e77260. doi: 10.2196/77260 (PMC12680938; doi:10.2196/77260)
Supplement: Multimedia Appendix 9 [file jmir_v27i1e77260_app9.docx]

**Multimedia Appendix 9.** Overall test results and between-group differences in expectant parental mindfulness level in the generalized estimating equations analysis.

| **Outcomes** | **Control group, (mean±SD)** | **Intervention group, (mean±SD)** | **Estimated mean difference,**  **mean (95% CI)** | ***Cohen's d*** | ***Group × time*** | |
| --- | --- | --- | --- | --- | --- | --- |
|  |  |  |  |  | ***Waldχ^2^ (df)*** | ***P* value** |
| **Maternal mindfulness level** | |  |  |  |  |  |
| T1 | 62.23±7.90 | 62.79±7.79 | 0.56 (-1.85, 2.98) |  | 13.611 | <0.001 |
| T2 | 61.68±6.91 | 67.31±9.42 | 5.54 (2.94, 8.15) | -0.68(0.36, 1.00) |  |  |
| **Paternal mindfulness level** | |  |  |  |  |  |
| T1 | 62.81±9.13 | 61.60±9.34 | -1.21 (-4.06, 1.63) |  | 4.738 | 0.030 |
| T2 | 63.51±9.16 | 66.05±10.95 | 2.48 (-0.74, 5.70) | -0.25(0.00, 0.57) |  |  |

T1: baseline (12 to 20 weeks of gestation); T2: two weeks after the completion of intervention (approximately 20 to 28 weeks of gestation).
