# Supplementary material for: Effects of Digital Mindfulness Training for Couples on Psychological Distress and Infant Neuropsychological Development: Randomized Controlled Trial
Source: J Med Internet Res. 2025 Nov 21;27:e77260. doi: 10.2196/77260 (PMC12680938; doi:10.2196/77260)
Supplement: Multimedia Appendix 10 [file jmir_v27i1e77260_app10.docx]

**Multimedia Appendix 10.** Correlation between expectant parental psychological distress during pregnancy and infant temperament.

| **parental psychological distress (T2)** | **Activity** | **Approach** | **Adaptability** | **Intensity of reaction** | **Quality of mood** | **Distractibility** |
| --- | --- | --- | --- | --- | --- | --- |
| Maternal depression ^a^ | 0.112 | 0.237^*^ | 0.228^*^ | 0.219^*^ | 0.247^**^ | 0.254^**^ |
| Maternal perceived stress ^b^ | 0.163 | 0.239^*^ | 0.235^*^ | 0.316^**^ | 0.345^**^ | 0.318^**^ |
| Paternal depression ^c^ | 0.119 | -0.017 | 0.119 | 0.075 | 0.084 | 0.071 |

T2: two weeks after the completion of intervention, ^*^*P*<0.05, ^**^*P*<0.01, ^a^ Depression at baseline was adjusted for. ^b^ Perceived stress at baseline was adjusted for. ^c^ Fathers’ depression at baseline was adjusted for.
