# Supplementary material for: Effects of Digital Mindfulness Training for Couples on Psychological Distress and Infant Neuropsychological Development: Randomized Controlled Trial
Source: J Med Internet Res. 2025 Nov 21;27:e77260. doi: 10.2196/77260 (PMC12680938; doi:10.2196/77260)
Supplement: Multimedia Appendix 11 [file jmir_v27i1e77260_app11.docx]

**Multimedia Appendix** **11-1** The mediating role of maternal perceived stress (T2) between treatment allocation and infant approach.

|  | **Maternal Perceived Stress** | | | **Infant Approach** | | | **Infant Approach** | | |
| --- | --- | --- | --- | --- | --- | --- | --- | --- | --- |
|  | *B* | *SE* | *P* | *B* | *SE* | *P* | *B* | *SE* | *P* |
| **Treatment Allocation** | -4.289 | 0.925 | <0.001 | -0.317 | 0.169 | 0.063 | -0.169 | 0.182 | 0.354 |
| **Maternal** **Perceived Stress** |  |  |  |  |  |  | 0.034 | 0.017 | 0.043 |
| ***R^2^*** | 0.358 | | | 0.088 | | | 0.121 | | |
| ***F*** | 32.090^***^ | | | 5.567^**^ | | | 5.211^**^ | | |

T2: Two weeks after the completion of intervention; ^**^*P*＜0.01, ^***^*P*＜0.001; The mediational analysis controlled for maternal Perceived Stress at T1.

**Multimedia Appendix** **11-2** The mediating role of maternal perceived stress (T2) between treatment allocation and infant reaction intensity.

|  | **Maternal Perceived Stress** | | | **Infant** **Reaction Intensity** | | | | **Infant Reaction Intensity** | | |
| --- | --- | --- | --- | --- | --- | --- | --- | --- | --- | --- |
|  | *B* | *SE* | *P* | *B* | *SE* | *P* | *B* | | *SE* | *P* |
| **Treatment Allocation** | -4.289 | 0.925 | <0.001 | -0.502 | 0.178 | 0.006 | -0.301 | | 0.189 | 0.114 |
| **Maternal Perceived Stress** |  |  |  |  |  |  | 0.047 | | 0.018 | 0.009 |
| ***R^2^*** | 0.358 | | | 0.077 | | | 0.131 | | | |
| ***F*** | 32.090^***^ | | | 4.802^*^ | | | 5.745^**^ | | | |

T2: Two weeks after the completion of intervention; ^**^*P*＜0.01，^***^*P*＜0.001; The mediational analysis controlled for maternal perceived stress at T1.

**Multimedia Appendix 11-3** The mediating role of maternal perceived stress (T2) between treatment allocation and infant quality of mood

|  | **Maternal Perceived Stress** | | | **Infant Quality of Mood** | | | **Infant Quality of Mood** | | |
| --- | --- | --- | --- | --- | --- | --- | --- | --- | --- |
|  | *B* | *SE* | *P* | *B* | *SE* | *P* | *B* | *SE* | *P* |
| **Treatment Allocation** | -4.289 | 0.925 | <0.001 | -0.563 | 0.119 | <0.001 | -0.441 | 0.127 | 0.001 |
| **Maternal Perceived Stress** |  |  |  |  |  |  | 0.028 | 0.012 | 0.017 |
| ***R^2^*** | 0.358 | | | 0.176 | | | 0.216 | | |
| ***F*** | 32.090^***^ | | | 12.252^***^ | | | 10.452^***^ | | |

T2: Two weeks after the completion of intervention; ^**^*P*＜0.01，^***^*P*＜0.001; The mediational analysis controlled for maternal perceived stress at T1.

**Multimedia Appendix 11-4** The mediating role of maternal psychological stress symptoms (T2) between treatment allocation and infant distractibility.

|  | **Maternal Perceived Stress** | | | **Infant** **Distractibility** | | | **Infant Distractibility** | | |
| --- | --- | --- | --- | --- | --- | --- | --- | --- | --- |
|  | *B* | *SE* | *P* | *B* | *SE* | *P* | *B* | *SE* | *P* |
| **Treatment Allocation** | -4.289 | 0.925 | <0.001 | -0.589 | 0.151 | <0.001 | -0.440 | 0.162 | 0.007 |
| **Maternal Perceived Stress** |  |  |  |  |  |  | 0.035 | 0.015 | 0.022 |
| ***R^2^*** | 0.358 | | | 0.149 | | | 0.187 | | |
| ***F*** | 32.090^***^ | | | 10.069^***^ | | | 8.755^***^ | | |

T2: Two weeks after the completion of intervention; ^**^*P*＜0.01，^***^*P*＜0.001; The mediational analysis controlled for maternal perceived stress at T1.
